# Age-related self-DNA accumulation may accelerate arthritis in rats and in human rheumatoid arthritis

**DOI:** 10.1038/s41467-023-40113-3

**Published:** 2023-07-20

**Authors:** Wei-Dan Luo, Yu-Ping Wang, Jun Lv, Yong Liu, Yuan-Qing Qu, Xiong-Fei Xu, Li-Jun Yang, Zi-Cong Lin, Lin-Na Wang, Rui-Hong Chen, Jiu-Jie Yang, Ya-Ling Zeng, Rui-Long Zhang, Bai-Xiong Huang, Xiao-Yun Yun, Xuan-Ying Wang, Lin-Lin Song, Jian-Hui Wu, Xing-Xia Wang, Xi Chen, Wei Zhang, Hui-Miao Wang, Li-Qun Qu, Meng-Han Liu, Liang Liu, Betty Yuen Kwan Law, Vincent Kam Wai Wong

**Affiliations:** 1 Dr. Neher’s Biophysics Laboratory for Innovative Drug Discovery, Macau University of Science and Technology, Macau, China; 2 The Affiliated Hospital of Southwest Medical University, Southwest Medical University, Luzhou, 646000 China; 3 State Key Laboratory of Quality Research in Chinese Medicine, Macau University of Science and Technology, Macau, China; 4 The Affiliated Traditional Chinese Medicine Hospital of Southwest Medical University, Southwest Medical University, Luzhou, 646000 China

**Keywords:** Mechanisms of disease, Rheumatoid arthritis, Ageing, DNA damage and repair

## Abstract

The incidence of rheumatoid arthritis (RA) is increasing with age. DNA fragments is known to accumulate in certain autoimmune diseases, but the mechanistic relationship among ageing, DNA fragments and RA pathogenesis remain unexplored. Here we show that the accumulation of DNA fragments, increasing with age and regulated by the exonuclease TREX1, promotes abnormal activation of the immune system in an adjuvant‐induced arthritis (AIA) rat model. Local overexpression of TREX1 suppresses synovial inflammation in rats, while conditional genomic deletion of TREX1 in AIA rats result in higher levels of circulating free (cf) DNA and hence abnormal immune activation, leading to more severe symptoms. The dysregulation of the heterodimeric transcription factor AP-1, formed by c-Jun and c-Fos, appear to regulate both TREX1 expression and SASP induction. Thus, our results confirm that DNA fragments are inflammatory mediators, and TREX1, downstream of AP-1, may serve as regulator of cellular immunity in health and in RA.

## Abstract (web summary)

The incidence of rheumatoid arthritis (RA) and accumulation of circulating free (cf) DNA increase with age but it is unknown whether DNA fragments cause joint inflammation. Here authors show that cf DNA levels are higher in RA patients and that in a rat adjuvant-induced arthritis model, the exonuclease TREX1 suppresses synovial inflammation via promoting the degradation of cf DNA and inhibiting a senescence-like cellular state.

## Introduction

Rheumatoid arthritis (RA) is a chronic and systemic autoimmune disease with an unclear pathogenesis^1^. Current studies have suggested that multiple factors, including ageing, a smoking history, immunogenicity, and genetic polymorphisms, may be associated with the pathogenesis of RA and its refractory problems. Notably, treatment with the current DMARDs is often associated with serious side effects and the development of drug resistance. With the disease progression time of 5–10 years and a disability rate of ~43–48%^2^, effective prevention or delay of the onset of RA would be of great benefit to RA patients. Therefore, the identification and characterization of genes related to the onset of RA may lay a solid theoretical foundation for the development of preventive therapeutic approaches for RA in the future.

Ageing is currently an inevitable process for humans, and age-associated chronic diseases are the predominant contributors to global morbidity, hospitalization, health care costs and mortality^3^. Epidemiological studies have identified a variety of age-related diseases, including atherosclerosis, Alzheimer’s disease, osteoarthritis, cancer, type 2 diabetes and RA^4–6^. Although RA showed a global prevalence of approximately 1%^7^, more than 50% of middle-aged and elderly adults were reported to develop RA in the United States between 2004 and 2014^8^. Thus, studies on the causes of the high incidence of RA in elderly people are necessary to establish preventive strategies for RA.

Studies have shown that metabolic DNA damage and sustained activation of the immune system are important biological activities that drive cellular ageing^9^. During the ageing process, DNA fragments accumulate and circulate in the bloodstream to become circulating free DNA (cfDNA)^10^. Of note, DNA fragmentation is also considered a biomarker for many diseases, including trauma, sepsis, aseptic inflammation, myocardial infarction, stroke, transplant rejection, diabetes mellitus and sickle cell disease^11^. In fact, both Aicardi-Goutières syndrome and familial frostbite lupus occur with a common phenotypic feature of failure to properly clear self-DNA or self-RNA fragments, leading to abnormal activation of the immune system^12^. Intriguingly, RA patients treated with disease-modifying anti-rheumatic drugs (DMARDs) were found to have a significant decrease in plasma DNA fragmentation^13^, indicating the possible correlation of DNA fragmentation with the pathogenesis of RA. Based on the hypothesis that the increased incidence of RA in middle-aged and elderly patients may be due to age-related accumulation of DNA fragments, determining the origin and promoting the clearance mechanisms of plasma DNA fragments in these patients may open a new avenue for developing prevention and treatment strategies for RA.

In the present study, the accumulation of circulating DNA fragments in the AIA model was confirmed to be triggered by reduced expression of TREX1, which eventually promoted the release of proinflammatory cytokines and generated inflammatory conditions in AIA rats. With validation in conditional knockout rats via the Cre-LoxP system, the current study revealed that knockdown of the 3’–>5’ DNA exonuclease TREX1 increased the severity of arthritis in AIA rats. In contrast, overexpression of TREX1 by adeno-associated virus (AAV) transduction significantly inhibited the senescence-associated secretory phenotype (SASP) and suppressed arthritic inflammation in AIA rats. Further mechanistic studies revealed that a decrease in c-Fos (a major component of TREX1 transcription factor AP-1) in senescent cells was accompanied by a reduction of sensitivity toward DNA fragments stimulation, which contributed to a drop of TREX1 transcript levels, and maybe leading to the prolonged inflammatory activation. Our findings highlight that the accumulation of DNA fragments due to reduced TREX1 may be one of the risk factors for the pathogenesis of RA in the elderly.

## Results

### The 3’->5’ DNA exonuclease TREX1 is significantly downregulated in RA patients

**Fig. 1:**
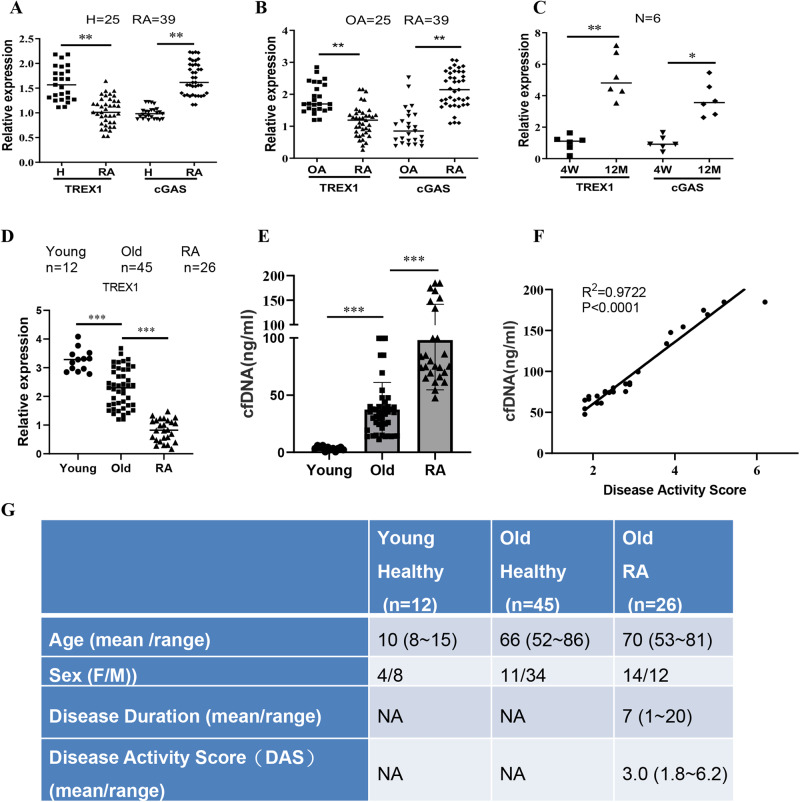
Correlation of TREX1 and cGAS expression in the production of inflammatory cytokines in rheumatoid arthritis (RA) patients and RA-FLSs stimulated with DNA fragments. **A** Gene expression levels of TREX1 and cGAS in healthy volunteers (*n* = 25) versus patients with RA (*n* = 39). **B** Gene expression levels of TREX1 and cGAS in patients with osteoarthritis (OA) (n = 25) versus patients with RA (*n* = 39). The values in the bar chart are the means ± s.e.m.; **P < 0.01. **C** Gene expression levels of TREX1 and cGAS in peripheral blood samples from young (4 weeks) and aged (>12 months) rats (*n* = 6). **D** The mRNA expression levels of TREX1 and (**E**) the cfDNA concentrations in the peripheral blood serum of healthy children (*n* = 12), healthy elderly (*n* = 45), and elderly patients with RA (*n* = 26). **F** Correlative analysis of disease activity scores (DAS) of elderly patients with RA and their serum cfDNA concentrations. In the correlative analysis, results shows a significant medium positive correlation between DAS and serum cfDNA concentrations in which the correlation coefficient was derived as 0.9722 and *p* < 0.0001. **G** Detailed information of clinical samples used in (**D**~**F**). All the samples are biologically independent, and in A-E, the statistical significance was calculated by *t*-test, **P < 0.01, ***P < 0.001. Da*t*a are presented as mean ± s.e.m.

Ageing is considered an important risk factor for RA^14^ via unknown mechanisms. In this study, we hypothesized that cfDNA-mediated activation of autoimmunity and dysfunction of DNA clearance enzymes may be responsible for the progression and development of RA in elderly individuals. To investigate whether the accumulation of DNA fragments can stimulate an abnormal immune response, we first determined the gene expression levels of TREX1 (a DNA fragment clearance enzyme) and cGAS (a DNA fragment sensor) in peripheral blood samples from healthy volunteers and RA and osteoarthritis (OA) patients. As shown in Fig. 1A, B, the gene expression level of TREX1 was significantly reduced but the level of cGAS was markedly increased in RA patients compared with healthy controls (*P* < 0.01) and patients with OA (*P* < 0.01), suggesting that TREX1 is closely associated with the pathogenesis of RA in humans. Consistent with the observation that the gene expression of TREX1 is associated with the clearance of cytoplasmic DNA fragments and ageing^15^, Figure 1C shows that the transcription levels of both TREX1 and cGAS were highly elevated in blood samples from middle age healthy rats (>12 months old) compared to young healthy rats (4 weeks old). To investigate the relationship between TREX1 expression levels and the cfDNA concentrations in the clinic, the mRNA expression levels of TREX1 and cfDNA concentrations in human peripheral blood serum samples from healthy children, healthy elderly, and elderly patients with RA were determined. As shown in Fig. 1D, the TREX1 mRNA expression level of healthy elderly and elderly RA patients were much lower compared with that in healthy children. Notably, the elderly patients with RA showed an even lower expression of TREX1 in comparison with healthy elderly. Simultaneously, the serum cfDNA concentrations of these three groups were measured and shown in Fig. 1E. Of note, the concentration of serum cfDNA from healthy elderly and elderly patients with RA demonstrated a significant increase compared with that in healthy children. In addition, the elderly patients with RA showed an even higher cfDNA accumulation in comparison with healthy elderly. The results in Fig. 1D, E indicated that aging and the onset of RA are accompanied by the reduction of TREX1 expression and the accumulation of cfDNA in blood serum. Coincidentally, as the substrate of TREX1, the elevation of cfDNA showed a contrary trend to TREX1 reduction, indicating that the reduction of TREX1 could be a risk factor for the onset of RA in elderly and may exacerbate the pathogenesis of RA *via* accumulating serum cfDNA concentration. To determine whether cfDNA will exacerbate the pathology of RA, a correlative analysis of RA disease activity scores 28 (DAS28) of elderly RA patients and their serum cfDNA concentrations were evaluated. As illustrated in Fig. 1F, the severity of RA pathology showed a good relevance to the increment of serum cfDNA concentration. Collectively, results in Fig. 1D–F indicated that both TREX1 downregulation and serum cfDNA accumulation may increase the risk of RA onset and exacerbate the disease severity of RA. The detailed information of clinical samples was shown in Fig. 1G. These findings may indicate that the level of cytoplasmic DNA fragments increases with age and that the accumulation of DNA fragments may activate the cGAS signalling cascade. In addition, a high level of TREX1 expression is required for the clearance excessive DNA fragments from middle age rats, which may prevent an abnormal immune response and maintain the health status of animals.

### Role of TREX1 and cGAS signalling in DNA fragment-induced release of proinflammatory cytokines

**Fig. 2:**
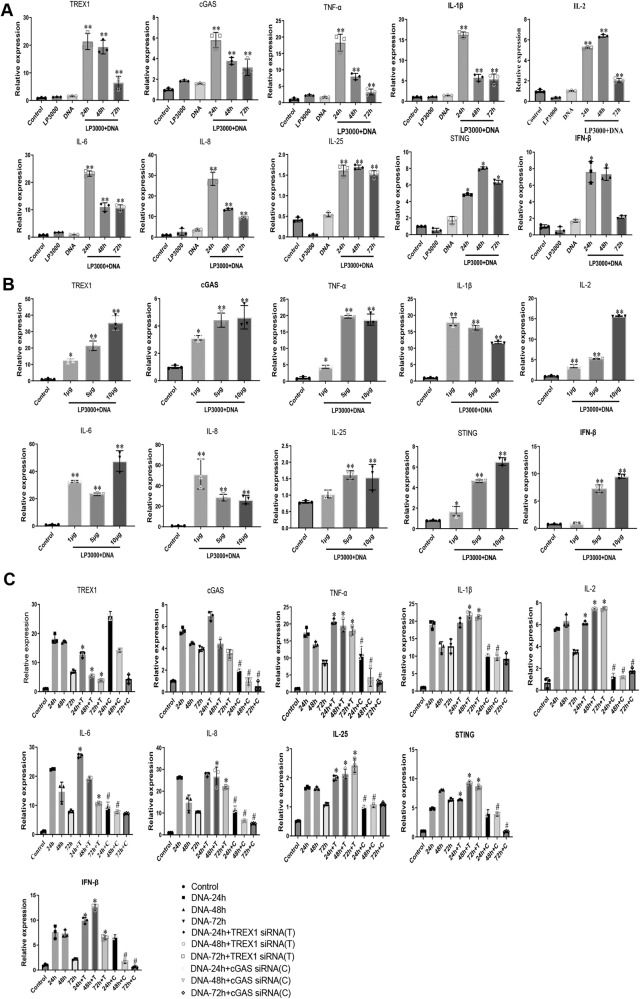
Gene expression analysis of TREX1, cGAS and inflammatory cytokines in DNA-stimulated RA-FLSs transfected with or without TREX1 or cGAS si*RNA*. **A** Time-dependent activation of the TREX1 and cGAS signalling pathways induced by DNA fragments. RA-FLSs were transfected with 5 μg of DNA fragments (sonicated DNA from RA-FLSs) for 24–72 h in the absence or presence of Lipofectamine 3000. Transfected RA-FLSs were then harvested for gene expression analysis of TREX1, cGAS and inflammatory cytokines using RT–PCR. All samples are biologically independent, and statistical significance was analyzed by one-way ANOVA, *P < 0.05, **P < 0.01 versus control groups. Data are presented as the mean ± s.e.m from three independent experiments. **B** Dose-dependent activation of the TREX1 and cGAS signaling pathways by DNA fragments. RA-FLSs were transfected with different amounts of DNA fragments (1 μg, 5 μg and 10 μg) in the presence of Lipofectamine 3000 for 24 h. Transfected RA-FLSs were then harvested for gene expression analysis of TREX1, cGAS and inflammatory cytokines using RT–PCR. All samples are biologically independent, and statistical significance was calculated by one-way ANOVA, *P < 0.05, **P < 0.01 versus control groups. Data are presented as the mean ± s.e.m. from three independent experiments. **C** Knockdown of TREX1 or cGAS reversed the time-dependent activation of the TREX1 and cGAS signalling pathways in DNA fragment-stimulated RA-FLSs. RA-FLSs were transfected with TREX1 si*RNA* or cGAS siRNA for knockdown of the corresponding gene 24 h prior to treatment with DNA fragments. Knockdown cells were then transfected with 5 μg of DNA fragments for 24–72 h. Transfected cells were then harvested and analysed by RT–PCR to determine the mRNA expression of inflammatory cytokines. All samples are biologically independent, and statistical significance was calculated by one-way ANOVA, *P < 0.05 for TREX1 knockdown cells compared with cells transfected with DNA fragments alone; #*P* < 0.05 for cGAS knockdown cells compared with cells transfected with DNA fragments alone. Data are presented as mean ± s.e.m. from three independent experiments. (T: TREX1 siRNA; C: cGAS siRNA).

Upon UV-induced DNA damage, the transcription levels of TREX1 and the regulators of cGAS-STING signalling were significantly increased, with concomitant increases in the levels of proinflammatory cytokines such as TNF-α, IL-1β, IL-2, IL-6, IL-8, IL-25 and IFN-β in a UV exposure time-dependent manner (Supplementary Fig. 1A). Of note, silencing of TREX1 gene expression in RA-FLSs further enhanced the UV-mediated production of these proinflammatory cytokines, whereas knockdown of cGAS significantly suppressed the UV-mediated release of these proinflammatory cytokines. These findings indicate that both TREX1 and cGAS-STING signalling participate in the regulation of DNA damage response (DDR)-induced inflammatory responses. The UV-mediated DDR can result in the production of DNA fragments that induce multiple intracellular reactions^16^ and inflammatory responses. Therefore, sheared DNA fragments (sonicated DNA from RA-FLSs) were transfected into RA-FLSs to investigate whether the accumulation of DNA fragments can induce an inflammatory response (Supplementary Fig. 1B). As shown in Fig. 2A, B, transfection of DNA fragments into RA-FLSs stimulated the expression of the DNA fragment clearance enzymes TREX1 and cGAS-STING, with concomitant upregulation of proinflammatory cytokines, in a dose-dependent manner. However, it was found that DNA fragments increased the expression of TREX1, cGAS-STING and proinflammatory cytokines only within the first 24 h and that the expression of these activated genes then decreased from 48 to 72 h post-transfection. Therefore, it was postulated that DNA fragment-induced upregulation of TREX1 may facilitate the clearance of the accumulated cytoplasmic DNA fragments and reduce the levels of cGAS-STING and proinflammatory cytokines. Consistent with this hypothesis, the Western blot results also confirmed that the addition of DNA fragments into RA-FLSs increased the expression of the TREX1 and cGAS proteins in a time- and dose-dependent manner (Supplementary Fig. 1C). To reveal the role of TREX1 and cGAS-STING signalling in the DNA fragment-induced inflammatory response, the TREX1 and cGAS were silenced in RA-FLSs with siRNA prior to challenge with DNA fragments. As shown in Fig. 2C and Supplementary Fig. 2A, silencing of TREX1 gene expression activated cGAS-STING signalling and further promoted the release of proinflammatory cytokines in RA-FLSs challenged with DNA fragments, whereas knockdown of cGAS significantly downregulated cGAS-STING signalling and suppressed the DNA fragment-induced release of proinflammatory cytokines. These results confirmed that DNA fragments can stimulate inflammation and that both TREX1 and cGAS play a regulatory role in DNA fragment-mediated inflammatory responses.

### Knee joint cavity injection of DNA fragments promoted inflammatory responses in AIA rats

Prior to induction of arthritis in rats, joint injection of sonicated rat muscle DNA was performed in AIA rats to induce inflammatory responses. The pharmacokinetic study was conducted for determining the amount of circulating cfDNA in vivo, the DNA fragments (50, 100, and 200 μg) were tail-vein injected in healthy SD rats and their serum DNA fragment concentrations were then measured after 4–72 h. Results showed that their mean circulating cfDNA concentrations were around 50, 110, and 180 ng/ml at *T* = 72 h, respectively (Supplementary Fig. 2B). Based on our established AIA rat model, the concentration of cfDNA under pathological conditions is approximately 170 ng/ml (Supplementary Fig. 3). We therefore decided a joint injection concentration of DNA fragments with 200 μg and a tail vein injection concentration of DNA fragments with 100 μg. As shown in Supplementary Fig. 3A, B, immediate effects, such as severe swelling, erythema and joint rigidity, were identified in the knee joint cavity of AIA rats injected with 200 μg of DNA fragments. The drastic increases in the arthritis score and hind paw volume during Days 9–21 indicated that the arthritic conditions in AIA rats injected with DNA fragments were more severe than those in un-injected AIA rats. Moreover, the quantity of circulating free DNA (cfDNA) in serum was found to be significantly higher in both the un-injected AIA rats and the AIA rats injected with 50 or 200 μg of DNA fragments in comparison with the healthy control animals (Supplementary Fig. 3C). These findings revealed that a high level of cfDNA is commonly found in rats with AIA. Notably, the gene expression of TREX1 was significantly downregulated but that of the regulators of cGAS-STING signalling and proinflammatory cytokines such as TNF-ɑ, IL-1β, IL-2 and IL-6 was significantly upregulated in the peripheral blood of AIA rats with or without injection of DNA fragments (Supplementary Fig. 3D). Based on the observation that the injected DNA fragments may be cleared by TREX1 in the rat model prior to the onset of arthritis (Supplementary Fig. 3D), consistent with the gradual downregulation of TREX1 expression after the onset of AIA in rats, the hind paw volume and the arthritis scores in the AIA + DNA group were similar to those in the AIA group at Day 30. In addition, the proinflammatory effect of intravenous injection of DNA fragments on healthy rats was confirmed by the upregulation of TREX1 expression, the cGAS-STING signalling cascade and proinflammatory cytokine (TNF-ɑ, IL-1β, IL-2 and IL-6) expression for the first 48 h following injection (Supplementary Fig. 4). Therefore, tail vein injection of DNA fragments was performed at 48 h intervals after injection of complete adjuvant for the subsequent experiments (Supplementary Fig. 5-1A).

### Intravenous injection of DNA fragments exacerbated paw swelling and bone destruction in AIA rats

Based on the experimental conditions optimized in Supplementary Fig. 5-1A, the first symptom of hind paw swelling was observable on Day 12 in AIA rats intravenously injected with 100 μg of DNA fragments. This finding suggests that tail vein injection of DNA fragments induces the early onset of disease in AIA rats. Although no significant differences in the levels of proinflammatory cytokines were found in the peripheral blood of AIA rats when compared to that of AIA rats injected with DNA fragments (on Day 12) (Supplementary Fig. 5-1B), micro-CT analysis revealed a low bone mineral density (BMD), cortical mineral density (TMD), trabecular number, and total porosity in the swollen joints, indicating bone destruction in AIA rats injected intravenously with DNA fragments (Supplementary Fig. 5-2C). Apparently, the overall radiological and micro-CT scores demonstrated significant cartilage and bone destruction in AIA rats injected with 100 μg of DNA fragments when compared to rats treated with an equal volume of nucleic acid-free water or with 50 µg of DNA fragments (Supplementary Fig. 5-2D). Collectively, these results indicate that DNA fragment challenge may promote bone erosion in AIA rats.

### Joint injection of DNA fragments induced arthritis in TREX-1 conditional knockout (TREX1^Cre^) rats

**Fig. 3:**
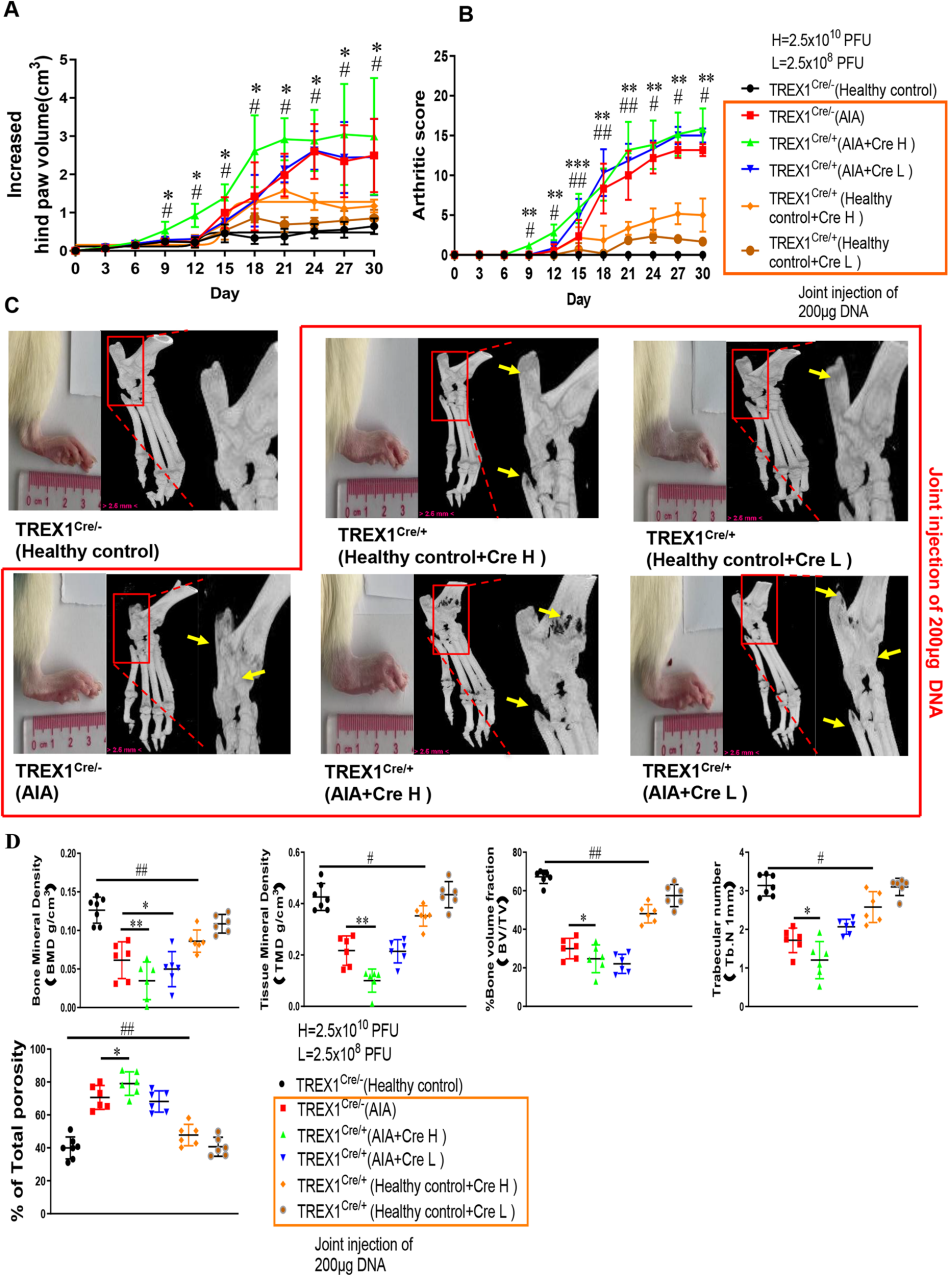
The effect of joint injection of DNA fragments on exacerbating inflammation in the TREX1 conditional knockout (TREX1^Cre^) rat AIA model. **A**, **B** Hind paw swelling and arthritis scores in DNA fragment-injected AIA model TREX1^Cre^ rats. Rats in the three healthy control TREX1^Cre^ groups and the three AIA model TREX1^Cre^ groups were injected in the knee joint cavity with nucleic acid-free water or DNA fragments (200 μg), respectively, together with Cre adeno-associated virus (2.5 × 10^8^ PFU (Cre L) or 2.5 × 10^10^ PFU (Cre H)), and monitored for 30 days. Hind paw volumes (ml) and arthritis scores were determined every 3 days. Hind paw swelling was photographed on Day 30. All samples are biologically independent, and statistical significance was calculated by *t*-test, #*P* < 0.05, ##*P* < 0.01 for healthy control TREX1^Cre/+^ rats with CreH/DNA compared with healthy control TREX1^Cre/-^ rats; *p < 0.05, **p < 0.01, ***p < 0.001 for AIA model TREX1^Cre/+^ rats with CreH/DNA compared with AIA model TREX1^Cre/-^ rats. Data are presented as the mean ± s.e.m. (Healthy control: *n* = 7, other groups: *n* = 6). **C** 3D Micro-CT images of damaged swollen joints bone were reconstructed using Inveon Research Workplace with a resolution of 19 μm. Various degrees of bone destruction are shown in the red box with enlarged images. The yellow arrows indicate the region of bone destruction. **D** Micro-CT scores of the bone destruction extent were calculated from five disease‐related indices: bone mineral density (BMD), trabecular number (mm-1) (Tb. N), cortical bone tissue mineral density (g/cm^3^) (TMD), bone volume fraction (BV/TV), and total porosity (as a percentage of total area). All the samples are biologically independent, and statistical significance was calculated by one-way ANOVA, #*p* < 0.05, ##*p* < 0.01 for healthy control TREX1^Cre/+^ rats with CreH/DNA compared with healthy control TREX1^Cre/-^ rats; *p < 0.05, **p < 0.01 for AIA model TREX1^Cre/+^ rats with CreH/DNA or CreL/DNA compared with AIA model TREX1^Cre/-^ rats. Data are presented as the mean ± s.e.m. (Healthy control: *n* = 7, other groups: *n* = 6).

To address the role of TREX1 in the pathogenic mechanism of DNA fragments in the AIA model, Cre-loxP-mediated conditional knockout of TREX1 was then accomplished in SD rats (TREX1^Cre^) by CRISPR/Cas-mediated genome editing. One week before AIA model establishment, TREX1^Cre^ rats were injected with 2 different doses of Cre adeno-associated virus (AAV; Cre-H: 2.5 × 10^10^ PFU, Cre-L: 2.5 × 10^8^ PFU), or vector control AAV in the joint cavity to knock down TREX1 gene expression. As shown in Fig. 3A–C, severe hind paw swelling, erythema and joint rigidity were found in the paws of both TREX1^Cre/+^ AIA rats and healthy control TREX1^Cre/+^ rats injected with 200 μg of DNA fragments in the knee joint cavity. The prompt increases in the arthritis score and hind paw volume further indicated that the arthritic condition was significantly exacerbated in AIA model TREX1^Cre/+^ rats in comparison with AIA rats after knee joint cavity injection of DNA fragments. Apparently, an arthritic condition was successfully induced in healthy control TREX1^Cre/+^ rats by knee joint cavity injection of DNA fragments, suggesting that knockdown of TREX1 may be a pathogenic risk factor for the development of RA. Micro-CT analysis further revealed severe joint swelling and bone destruction in DNA fragment-injected AIA model TREX1^Cre/+^ and healthy control TREX1^Cre/+^ rats by direct comparison of bone mineral density (BMD), cortical mineral density (Ct.TMD), bone volume fraction, trabecular number, and total porosity to those in the healthy control group. Indeed, the AIA model TREX1^Cre/+^ rats injected with DNA fragments demonstrated the most severe joint swelling and cartilage erosion compared with healthy control TREX1^Cre/+^ rats (Fig. 3C, D). Furthermore, histopathological staining indicated that arthritic manifestations—for example, synovial cell hyperplasia with infiltration of neutrophils, lymphocytes and plasma cells; interstitial fibroblast proliferation accompanied by neovascularization; cartilage thickening in joint tissues; white pulp enlargement and fusion; lymphocyte proliferation in the spleen; thickening of the cortex and an increase in T lymphocytes in the thymus; glomerular oedema; and renal fibrosis—were concomitantly observed in the DNA fragment-injected AIA model TREX1^Cre/-^group, AIA model TREX1^Cre/+^ group and healthy control TREX1^Cre/+^ group relative to the healthy control group (Supplementary Figs. 6A and 7). In addition, immunohistochemical, protein and gene expression analyses revealed that in DNA fragment-injected AIA model TREX1^Cre/+^ rats, the lower the TREX1 expression level, the higher were the levels of proinflammatory factors and metalloproteinases (MMP-1, -3, -9) (Supplementary Figs. 6B and 8A, B). Collectively, these results indicate that knockdown or downregulation of TREX1 might increase the severity of the arthritic condition in control and AIA rats with knee joint cavity injection of DNA fragments. Our findings suggest that gene therapy of TREX1 may provide a beneficial therapeutic effect in RA, possibly through the clearance of cfDNA in joint tissues.

### DNA fragments promoted T-cell activation and decreased Foxp3 cell populations in TREX1^Cre^ rats

**Fig. 4:**
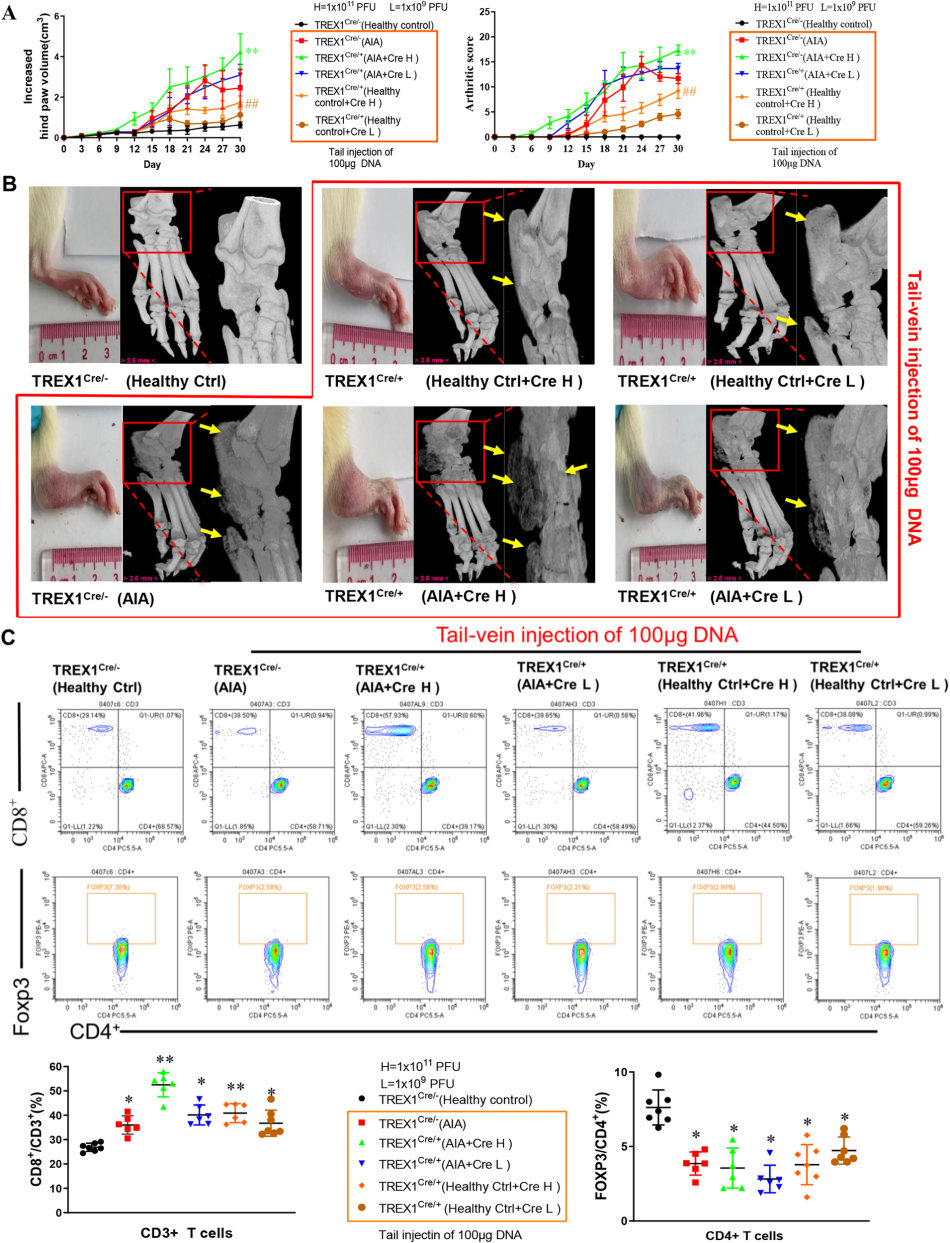
The effect of tail vein injection of DNA fragments on exacerbating inflammation in the TREX1^Cre^ rat AIA model. **A** Hind paw swelling and arthritis scores in AIA model TREX1^Cre^ rats with tail vein injection of DNA fragments. All the samples are biologically independent, and statistical significance was calculated by *t*-test, ##*P* < 0.01 for healthy control+CreH group compared with the healthy control group; **P < 0.01 for AIA +CreH group compared with AIA group. Data are presented as the mean ± s.e.m. (Healthy control,Ctrl+Cre H, Ctrl+Cre L: *n* = 7,other groups: *n* = 6). **B** 3D Micro-CT images of damaged swollen joints’bone were reconstructed using Inveon Research Workplace with resolution of 19 μm. Various degrees of bone destruction are shown in the red box with enlarged images. The yellow arrows indicate the region of bone destruction (Healthy control, Ctrl+Cre H, Ctrl+Cre L: *n* = 7, other groups: *n* = 6). **C** Immunological effect of tail vein injection of DNA fragments in AIA model TREX1^Cre^ rats. After the rats in the three healthy control TREX1^Cre^ groups and the three AIA model TREX1^Cre^ groups were injected via the tail vein with DNA fragments (100 μg) together with Cre adeno-associated virus (1 × 10^9^ PFU (Cre L) or 1 × 10^11^ PFU (Cre H)) and monitored for 30 days, blood lymphocytes were harvested from these animals for flow cytometric analysis of T-cell activation using fluorescent antibodies against CD45, CD3, CD4, CD8, and Foxp3. Representative flow charts for the purification of CD8^+^ cells gated on CD3^+^ T lymphocytes and Foxp3 cells gated on CD4^+^ T lymphocytes from Peyer’s patches (PPs). The quantitative bar charts show the percentage of CD8^+^ T cells amsong CD3^+^ T cells and the percentage of Foxp3^+^ Treg cells among CD4^+^ T cells. All the samples are biologically independent, and statistical significance was calculated by one-way ANOVA, *p < 0.05, **p < 0.01 versus the healthy control group. Data are presented as the mean ± s.e.m. (Healthy control, Ctrl+Cre H, Ctrl+Cre L: *n* = 7, other groups: *n* = 6).

Systemic immune abnormalities are one of the key pathogenic factors for RA development^17^. In this study, we evaluated whether DNA fragment injection can stimulate T-cell activation in TREX1^Cre^ rats. To examine the autoimmune responses induced by DNA fragment challenge, sonicated rat muscle DNA fragments were injected into both healthy control TREX1^Cre/+^ rats and AIA model TREX1^Cre/+^ rats via the tail vein. Consistent with the above results, more severe hind paw swelling, a significantly higher arthritis score, and severe bone destruction were observed in the swollen joints of healthy control TREX1^Cre/+^ rats and AIA model TREX1^Cre/+^ rats injected with DNA fragments (Fig. 4A, B). On Day 30, both CD4^+^ and CD8^+^ T lymphocytes were separated and purified from total peripheral blood lymphocytes with antibodies against CD3^+^. As shown in Fig. 4C, flow cytometry analysis showed that the CD8^+^ T lymphocyte population was significantly increased but the Foxp3 cell population was markedly reduced in AIA model TREX1^Cre/-^ rats in comparison with healthy control rats. Of note, tail vein injection of DNA fragments further increased the number of CD8^+^ T lymphocytes and decreased the Foxp3 cell population in both AIA model TREX1^Cre/+^ (Cre H/L) rats and healthy control TREX1^Cre/+^ (Cre H/L) rats compared with healthy control TREX1 wild-type rats (Fig. 4C). Accordingly, both lower gene expression of TREX1 and accumulation of DNA fragments might be the crucial participants in the aberrant activation of immune cells during the pathogenesis of RA.

### Adenovirus (AAV)-mediated overexpression of TREX1 suppressed synovial inflammation in AIA rats

**Fig. 5:**
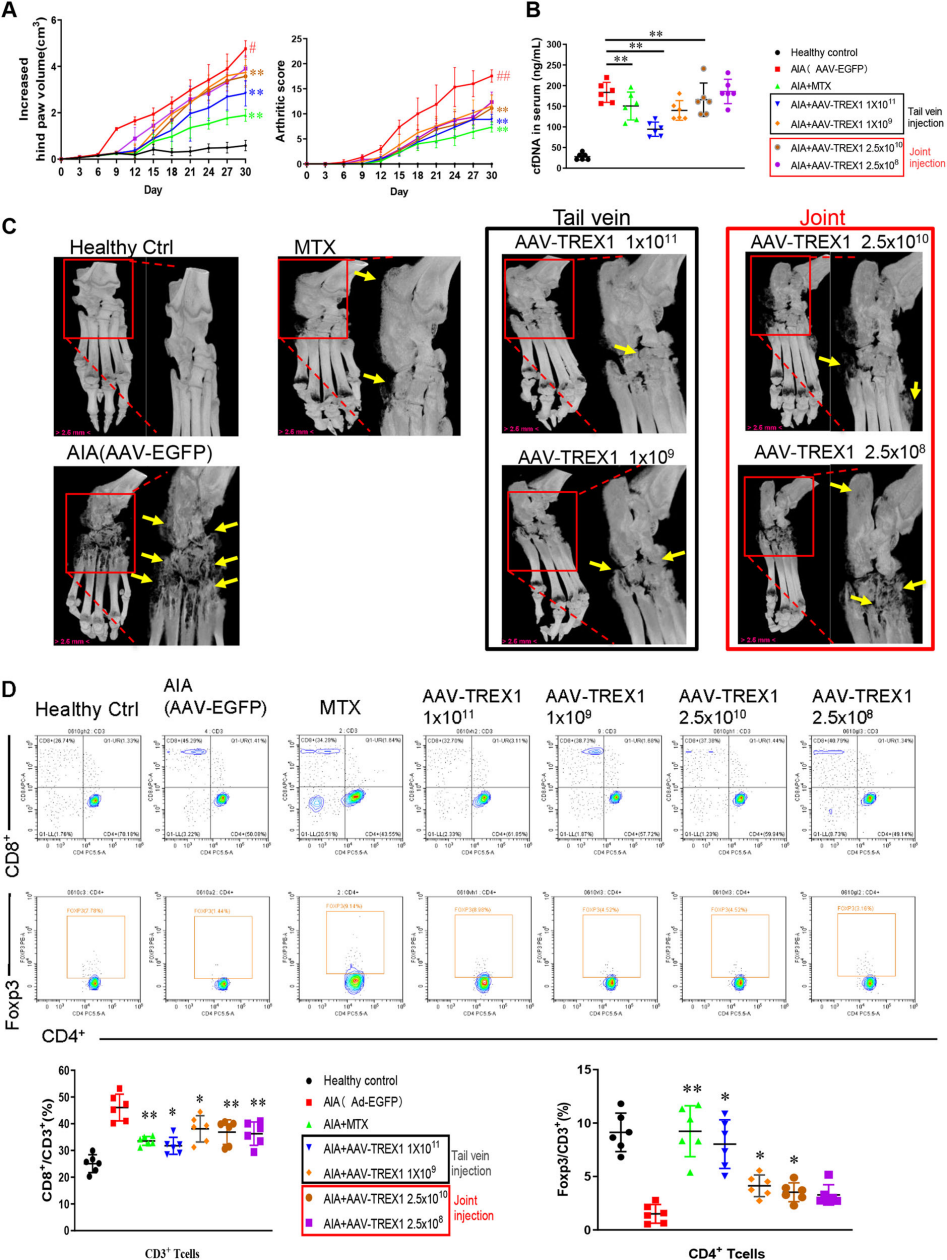
The anti-inflammatory role of TREX1 in AIA rats. **A** Hind paw volumes and arthritis scores in AIA rats with AAV-mediated TREX1 overexpression. Healthy control and AIA rats were treated with vehicle control, the positive control drug (7.6 mg/kg/week MTX), AAV-TREX1 (1 × 10^11^ or 1 × 10^9^ PFU) by tail vein injection 10 days before AIA induction or with AAV-TREX1 (2.5 × 10^10^ or 2.5 × 10^8^ PFU) by joint injection 10 days before AIA induction. Arthritis scores and hind paw volumes were measured every 3 days. All the samples are biologically independent, and statistical significance was calculated by one-way ANOVA, **p < 0.01 versus the AIA group, #*p* < 0.05, ##*p* < 0.01 for the AIA group compared with the healthy control group. Data are presented as the mean ± s.e.m. (*n* = 6 in all groups). **B** Changes in the cfDNA concentration in the serum of AIA rats with AAV-mediated TREX1 overexpression. On Day 30, the amount of cfDNA in serum from all treatment groups was measured. The amount of cfDNA in each treatment group was quantified as well. All samples are biologically independent, and statistical significance was calculated by one-way ANOVA, **p < 0.01 versus the AIA group. Data are presented as the mean ± s.e.m. (All groups *n* = 6). **C** 3D Micro-CT images of damaged swollen joints’bone were reconstructed using Inveon Research Workplace with a resolution of 19 μm. Various degrees of bone destruction are shown in the red box with enlarged images. The yellow arrows indicate the region of bone destruction. **D** Immunomodulatory effect of TREX1 in AIA rats. For immunological analysis, blood lymphocytes were harvested from these animals for flow cytometric analysis of T-cell activation using fluorescent antibodies against CD45, CD3, CD4, CD8, and Foxp3. Representative flow charts for the purification of CD8^+^ cells gated on CD3^+^ T lymphocytes and Foxp3 cells gated on CD4^+^ T lymphocytes from Peyer’s patches (PPs). The quantitative bar charts show the percentage of CD8^+^ T cells among CD3^+^ T cells and the percentage of Foxp3^+^ Treg cells among CD4^+^ T cells. All samples are biologically independent, and statistical significance was calculated by one-way ANOVA, *p < 0.05, **p < 0.01 versus the AIA group. Data are presented as the mean ± s.e.m. (All groups *n* = 6).

To further investigate the role of TREX1 in the pathogenesis of AIA in rats, adeno-associated virus expressing TREX1 (AAV-TREX1) was injected into the joint cavity (2.5 × 10^10^ PFU or 2.5 × 10^8^ PFU) or tail vein (1.0 × 10^11^ PFU or 1.0 × 10^9^ PFU) of rats. As shown in Fig. 5A, severe swelling, erythema and joint rigidity were found in the hind paws of AIA rats injected with AAV-EGFP (vehicle control). In contrast, similar to MTX-treated rats, AIA rats injected with AAV-TREX1 in either the knee joint cavity or tail vein showed significant decreases in the arthritis score and hind paw volume, suggesting that the severity of AIA was markedly attenuated in rats with overexpression of TREX1. On Days 9 and 12, significant differences in the arthritis score were observed between AAV-TREX1- and AAV-EGFP-injected AIA rats. Extensive accumulation of DNA fragments in serum was apparent in AAV-EGFP-injected AIA rats in comparison with healthy control rats. Importantly, joint cavity or tail vein injection of AAV-TREX1 markedly reduced the serum concentration of DNA fragments in AIA rats (Fig. 5B), indicating that TREX1 inhibits joint swelling possibly *via* clearance of DNA fragments. Micro-CT analysis of hind paws further demonstrated the beneficial effects of TREX1 in AIA rats (Fig. 5C), and the protective effects on bone cartilage were consistent with the decreasing trends in the arthritis score and hind paw swelling in AIA rats with TREX1 overexpression. Collectively, these findings indicate that TREX1 might play a suppressive role in the development of arthritis in AIA rats *via* the clearance of DNA fragments.

### The AAV-TREX1 suppressed immune response in AIA rats may be correlated to the activation of the Foxp3 cell population

Given that that reduction in TREX1 expression induced an arthritic phenotype through DNA fragment accumulation and aberrant T-cell activation in the AIA and TREX1^Cre/+^ models, whether TREX1 overexpression can reverse the DNA fragment-induced imbalance in the T lymphocyte ratio was further investigated. First, T lymphocytes were isolated from the peripheral blood of AIA rats with or without injection of AAV-TREX1 on Day 28. As shown in Fig. 5D, flow cytometry analysis showed that while the CD8^+^ T lymphocyte population was significantly increased, the Foxp3 cell population was significantly decreased in the AAV-EGFP-injected AIA group. In contrast, adenovirus-induced overexpression of TREX1 in AIA rats markedly decreased CD8^+^ T lymphocytes and increased Foxp3 cells (Fig. 5D). Notably, Foxp3 cells were highly increased in AIA rats with tail vein injection of AAV-TREX1, suggesting that the abnormal immune response in AIA rats was suppressed by systemic overexpression of TREX1. Accordingly, TREX1 gene expression and DNA fragments might be potential targets for controlling the abnormal activation of immune cells during the pathogenesis of RA.

### Down-regulation of E2F1 and the concomitant drop of c-FOS after cellular senescence maybe responsible for the decreased sensitivity of regulating TREX1 expression after DNA stimulation, leading to the suppression of TREX1 expression

Based on the above in vivo experimental data, the SD rats in both the AIA and TREX1^Cre/+^ models exhibited the senescence-associated secretory phenotype (SASP), and the arthritis condition and ageing phenotype in these rats were reversed, together with reductions in the expression levels of proinflammatory cytokines (Supplementary Fig. 9A) and ageing markers^18^ such as IL-1β, IL-6, IL-8, IFN-β, p16 and p21 (Supplementary Fig. 9B and 10-1A), after MTX treatment or AAV-TREX1 injection. Therefore, decreased expression of TREX1 may be correlated with ageing during the onset of RA. Furthermore, direct comparison of TREX1 (Supplementary Fig. 9A) and cfDNA level (Fig. 5B) demonstrated that the TREX1 expression level was dose-dependently correlated with the injection doses of AAV-TREX1 in both the joint- and tail vein-injected AIA rats, but negatively correlated with the concentration of cfDNA (Supplementary Fig. 9C). The results indicated the modest changes in TREX1 level are responsible for the observed changes in DNA fragment level of AIA rats

**Fig. 6:**
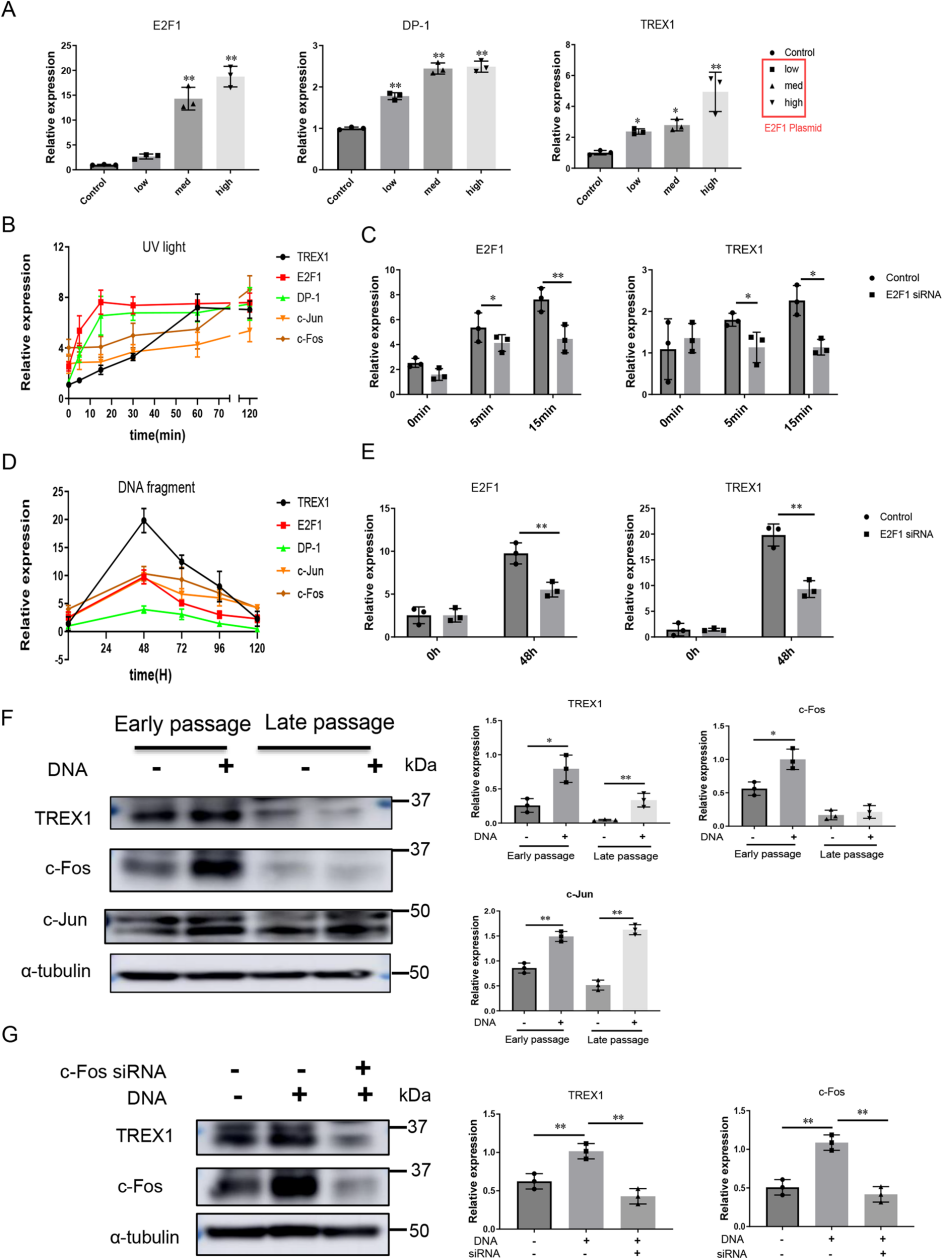
Transcriptional regulation of TREX1 via E2F1 signalling and the balance of c-Jun/c-Fos. **A** The expression levels of TREX1 and DP-1 in RA-FLSs overexpressing the transcription factor E2F1. RA-FLS were transfected with the E2F1 plasmid for 48 h. All samples are biologically independent, and statistical significance was calculated by one-way ANOVA, **P < 0.01 versus control groups. Data are presented as the mean ± s.e.m. from three independent experiments. **B** Gene expression profiles of TREX1, E2F1, DP-1, c-Jun and c-Fos in RA-FLSs exposed to UV light for different durations. **C** Role of E2F1 in the transcriptional regulation of TREX1 in UV-stimulated cells. RA-FLSs with siRNA-mediated E2F1 silencing were exposed to UV light for 0–15 min, and the gene expression levels of E2F1 and TREX1 were determined by RT–PCR. **D** Gene expression profiles of TREX1, E2F1, DP-1, c-Jun and c-Fos in RA-FLSs stimulated with DNA fragments. **E** Knockdown of E2F1 inhibited TREX1 activation in RA-FLSs transfected with DNA fragments. RA-FLSs with siRNA-mediated E2F1 silencing were transfected with 5 μg of DNA fragments for 48 h. **F** Presenescent RA-FLSs were rendered senescent by repeated passaging to late passages. Both early- and late-passage RA-FLSs were transfected with 5 μg of DNA fragments for 48 h. **G** Knockdown of c-Fos inhibited the activation of TREX1 in RA-FLSs transfected with DNA fragments. RA-FLSs with siRNA-mediated c-Fos silencing were transfected with 5 μg of DNA fragments for 48 h. In (**B**) and (**D**), all data were presented as the mean ± s.e.m. from three independent experiments. In (**C**) and (**E**), all samples are biologically independent, and statistical significance was calculated by *t*-test, *P < 0.05, **P < 0.01 between two groups. Data are presented as the mean ± s.e.m. from three independent experiments. In (**F**) and (**G**), the bar charts show the quantification of target protein expression relative to tubulin expression using ImageJ software. All samples are biologically independent, and statistical significance was calculated by *t*-test, **P < 0.01 between the two groups. Data are presented as the mean ± s.e.m. from three independent experiments. All samples are derived from the same experiment and the gels/blots were processed in parallel.

E2F1^15^ and AP-1^19^ are the key important transcription factors of TREX1. Consistent with this observation, our previous studies showed that both the mRNA and protein levels of E2F1 were significantly reduced in AIA rats and DNA fragment-challenged TREX1^Cre/+^ rats (Supplementary Fig. 10-1B, C); moreover, those of the AP-1 complex subunit c-Jun were increased but those of c-Fos were significantly decreased in various tissues from the same treatment groups (Supplementary Figs. 10-1B and 10-2A-C). Interestingly, our data showed that TREX1 expression was significantly increased in healthy SD rats after tail vein injection of DNA fragments and that the gene expression levels of c-Jun and c-Fos were also significantly increased in a time-dependent manner (Supplementary Fig. 10-2D). It suggests that the downregulation of TREX1in both AIA rats and DNA fragment-challenged TREX1^Cre/+^ rats was due to an imbalance in the c-Jun/c-Fos expression ratio. These findings were consistent with the data in Fig. 1C, which shows that increased expression of TREX1 is necessary for the clearance of excessive cfDNA from aged rats and DNA fragment-injected healthy rats. To further show the role of E2F1 in TREX1 expression, E2F1 was overexpressed dose-dependently in RA-FLSs, and the transcript levels of TREX1 and DP-1 (an important activator of the E2F transcription factor) were found to be significantly and gradually increased (Fig. 6A). Concomitantly, both UV-mediated DNA damage (Fig. 6B) and direct challenge with DNA fragments (Fig. 6D) induced the expression of E2F1, DP-1, c-Jun, and c-Fos with the upregulation of TREX1 in RA-FLSs in a time-dependent manner.

However, the increase in TREX1 expression was markedly suppressed in the presence of E2F1 siRNA (Fig. 6C, E and Supplementary Fig. 11), indicating that E2F1 is a key transcription factor of TREX1. To further elaborate on the regulatory role of TREX1 during ageing and RA, the expression of TREX1 and the AP-1 complex components (c-Jun/c-Fos) in both early- and late-passages RA-FLSs challenged with DNA fragments was determined. As shown in Fig. 6F, the expression of TREX1 in late-passages of RA-FLSs was significantly lower than that in early-passages RA-FLSs with or without DNA fragment challenge. Of note, c-Fos expression was induced in early-passages RA-FLSs challenged with DNA fragments but not in late-passages RA-FLSs. Furthermore, siRNA knockdown of c-Fos significantly inhibited DNA fragment-mediated TREX1 expression in early-passages RA-FLSs (Fig. 6G). In conclusion, all these findings suggest that both in ageing-related cellular senescence and in arthritic conditions, the expression of TREX1 is significantly downregulated via the suppression of its transcription factors, such as E2F1 and AP-1, these may further contribute to the accumulation of cfDNA, and eventually activate the autoimmune response. Accordingly, TREX1 dysregulation and cfDNA accumulation might be the risk factors promoting the progression and development of RA.

## Discussion

Ageing is an inevitable process in humans, and it has been associated with the onset of many diseases, including rheumatoid arthritis (RA)^20^. RA is a chronic, inflammatory systemic disease of unknown aetiology, predominantly affecting the synovium, that can lead to joint destruction and even disability in severe cases^1^. Notably, after the age of 50, the immune system undergoes dramatic changes, loses the ability to regulate immunity and gains proinflammatory functions. Accordingly, immunosenescence is particularly accelerated in patients with RA^21^. In fact, the ageing process contributes to cellular stress and eventually promotes apoptotic cell death and DNA fragment release^22^. Recent studies have reported that cell-free DNA (cfDNA) is frequently detected in the serum of individuals with advanced age^10^ and patients with cancer^23^, diabetes^24^, systemic lupus erythematosus (SLE)^25^, and many other diseases. Therefore, the accumulation of cfDNA or DNA fragments is considered a crucial biological marker for disease diagnosis and pathogenesis. Interestingly, our results demonstrated that the gene expression levels of TREX1 (a DNA fragment clearance enzyme) and cGAS (a DNA fragment sensor) in the peripheral blood samples of RA patients were significantly different from those in healthy volunteers. We also unravelled that TREX1 expression was markedly suppressed and cfDNA concentration was significantly elevated in elderly or elderly patients with RA in comparison with healthy children. Accordingly, our studies are the first to reveal the relationship between ageing-mediated cfDNA accumulation and TREX1 downregulation in the pathogenesis of RA.

In addition, we also confirmed that DNA fragments can stimulate the release of inflammatory factors through the cGAS pathway in two cellular models, such as UV-mediated DNA damaging model and DNA fragments accumulation model, in which the pro-inflammatory effect can be inhibited by overexpression of TREX1. Then we elaborated the relationship of RA pathogenesis with ageing by detecting in vivo cfDNA concentrations and injecting DNA fragments in adjuvant-induced arthritis (AIA) models. CfDNA fragments are well-characterized biomarkers for diseases and conditions such as ageing^10^, systemic lupus erythematosus^26^ and tumours^27^; accordingly, cfDNA may become one of the key factors in the pathogenesis of RA. Indeed, we successfully demonstrated the proinflammatory effect of DNA fragments in AIA rats and further revealed DNA fragment-induced premature joint swelling in AIA rats. We also firstly generated SD rats with Cre-LoxP-mediated conditional knockout of TREX1 (TREX1^Cre+/-^rats) and revealed that DNA fragments promoted proinflammatory cytokine release and T-cell activation in AIA rats when the DNA-metabolizing enzyme TREX1 was deleted, which is in line with in vitro studies showing that knockdown of TREX1 promoted the release of proinflammatory cytokines in RA-FLSs challenged with DNA fragments. Most importantly, analysis of blood serum from the AIA rat models revealed a significant increase in the DNA fragments concentration as well as a marked decrease in TREX1 expression. These findings suggested that DNA fragments may be a potential immunogenic factor for the induction of RA development. On the other hand, adeno-associated virus (AAV)-mediated overexpression of TREX1 in AIA rats was linked to a reduction in serum DNA fragments and a significant improvement in arthritis symptoms, for example, decreases in the arthritis score and hind paw swelling index, improvement in the micro-CT analysis index, and an increase in Foxp3 cells. Collectively, our data supported that TREX1 or DNA fragments may be the promising targets for RA treatment.

Accumulating evidence indicates that the secretion of inflammatory mediators is a distinctive feature of ageing^28^. For example, cell cycle arrest in senescent cells^29^, the loss of tissue repair capacity^30^, and the release of proinflammatory and matrix degradation factors^31^ are the common manifestations of the senescence-associated secretory phenotype (SASP)^32^. Notably, senescent cells exhibiting the SASP phenotype can promote various chronic diseases and conditions, including cardiovascular disease, metabolic syndrome, and neurocognitive decline, which are accompanied by the release of growth factors (such as TGF-α and IL-1), expression of transcription factors (such as GATA4 and NF-κB), and release of proinflammatory cytokines (such as IL-6 and IL-8), proteases and matrix metalloproteinases^33^. Numerous clinical studies of elderly individuals indicated that the levels of several cytokines, especially IL-6 and TNF-α, increase with age even in healthy individuals and in elderly individuals without acute infection; this characteristic is also termed inflammatory ageing^5^. Therefore, systemic inflammation is a developmental process related to ageing and ageing-related diseases. Inflammation is considered part of the normal repair response, and it is essential for the defence against bacterial and viral infections and harmful environmental factors^34^. However, the inflammatory process can also become destructive to organisms when it is prolonged and persistent. The SASP is a proinflammatory phenotype that accompanies mammalian ageing; however, the causative factors of this phenotype are not known^35^. IFN-β expression was found to be elevated in our DNA fragment-injected AIA model (Supplementary Fig. 3D); IFN-β is also a factor contributing to the SASP and has been reported to stimulate the DNA damage response (DDR) via the induction of ROS production^15^, which leads to senescence-like cell cycle arrest in human diploid fibroblasts (HDFs)^36^. IL-6, a common proinflammatory cytokine in RA, is another major factor contributing to the SASP; it can also increase intracellular ROS levels and cause DNA damage^37^. Importantly, antibodies against IL-6 or its receptor (tocilizumab) have become an effective targeted drugs prescribed for the clinical treatment of RA^38^. These findings indicate the strong correlations between ageing and RA. Intriguingly, our study demonstrated that the expression of the cell cycle activator E2F1^39^ was reduced but that of cell senescence-related genes such as p16 and p21^40^ was elevated in both the AIA and TREX1^Cre/+^ rat models, with concomitant elevation of senescence factors such as IL-1β, IL-6, IL-8 and IFN-β, suggesting that the SASP was induced in both AIA and TREX1^Cre/+^rats. Accordingly, we hypothesized that cell senescence-induced DNA fragmentation would increase the DDR and trigger positive feedback activation of DNA fragment accumulation, thereby accelerating the development of pathological ageing as well as the pathogenesis of RA. However, whether the downregulation of TREX1 in RA is a cause or a consequence of SASP induction requires further investigation in the future.

AP-1 is one of the transcription factors of TREX1 and is a dimeric complex composed of two important protein subunits, Jun and Fos^41^. The balance of Jun and Fos expression levels is important for the different transcriptional functions of AP-1, which include cell transformation^42^, angiogenesis^43^, and tumour invasion^44^. It has been shown that the expression of c-FOS is significantly reduced after fibroblast senescence, which leads to a decrease in TREX1 expression^19^. Consistent with these findings, we demonstrated that AP-1 exhibited elevated expression in wild-type rats after stimulation with DNA fragments, whereas it exhibited lower expression in TREX1^Cre^ rats and AIA rats owing to the imbalance in the c-Jun/c-Fos expression ratio. Accordingly, we speculated that the reason for the high incidence of RA in middle-aged and elderly patients may be due to the imbalance of Jun and Fos expression during ageing, resulting in a reduction in TREX1 expression. Consequently, the decreased expression of TREX1 may contribute to abnormal DNA metabolism and the proinflammatory effect of cfDNA accumulation, eventually driving normal cells to gradually acquire the SASP in inflammatory environments. Our results further confirmed that AAV-mediated overexpression of TREX1 markedly reversed the accumulation of cfDNA, suppressed the release of inflammatory cytokines, and ameliorated synovial tissue damage. Interestingly, we have identified that the serum cfDNA concentrations and the TREX1 expression were highly correlated with age in subsequent complementary clinical data, and further analysis shown that higher DAS28 (the Disease Activity Score using 28 joint counts, a score to evaluate disease activity in RA patients) scores were positively associated with higher cfDNA concentrations in blood serum of RA patients. Taken together, we are the first to demonstrate that DNA fragments could be a potential immunogenic factor for the induction of RA development, and the increase in serum cfDNA concentration, as well as the decrease in TREX1 expression, may be one of the clinical manifestations of premature organismal aging. Downregulation of TREX1 possibly contributes to the accumulation of DNA fragments in patients with RA, thereby further aggravating the inflammatory response *via* the activation of cGAS/STING signalling. Our findings may linkup autoimmune tissue inflammation to ageing and define effective clearance of DNA fragments as an important strategy for the management and prevention of RA.

## Methods

### Statement of ethics and regulations

Our clinical data collection was approved by the Research Ethics Committee of the Affiliated Hospital of Southwest Medical University (KY 2021010). The animal study was conducted according to the guidelines of the Institutional Animal Care and Use Committee of Macau University of Science and Technology and approved by the Ethics Committee of Macau University of Science and Technology (protocol code MUSTARE – 003 – 2020) on 23 Feb 2020.

### Cell culture and antibodies

The immortalized RA-FLS (MH7A) was purchased from Cell bank. Reagents for cell culture, such as Dulbecco’s modified Eagle’s medium (DMEM), foetal bovine serum (FBS), trypsin-EDTA (0.05%), phosphate-buffered saline (PBS), and 100× penicillin/streptomycin (PS), were obtained from Gibco (Oklahoma, USA). RA fibroblast-like synoviocytes (FLSs) were used between passages three and eight, and the cell type was identified as described in our previously published work. RA-FLSs were cultured in 6-well plates (1 × 10^5^ cells/well to ensure that the confluence reached 60–80%) in 2 ml of Dulbecco’s modified Eagle’s medium (DMEM) supplemented with 10% FBS and 1% PSG at 37 °C in a 5% CO_2_ humidified environment. The medium was removed and washed once with PBS for subsequent experiments. The anti-TREX1 antibody (diluted 1:1000 for WB and 1:200 for IHC)were purchased from Novus Biologicals, USA; the anti-cGAS antibody (1:1000 for WB) was obtained from Taiclone, China Taiwan; the anti-E2F1 antibody (1:1000), the anti-c-Jun antibody (1:1000 for WB) and anti-c-Fos antibody (1:1000 for WB) were obtained from Santa Cruz, USA, the anti-β-actin antibody (1:1000 for WB), the anti-GADPH antibody (1:1000 for WB) the anti-tubulin antibody (1:1000 for WB) were obtained from CST, USA, anti-rabbit secondary antibody (1:2500 for WB), and anti-mouse secondary antibody (1:2500 for WB) were purchased from Santa Cruz, USA; and flow cytometry antibodies such as APC/Cyanine7 anti-rat CD45 (1:1000), FITC anti-rat CD3 (1:1000), PerCP/Cyanine5.5 anti-rat CD4 (1:1000), APC anti-rat CD8a (1:1000) and PE anti-mouse/rat/human Foxp3 (1:1000) were purchased from Biolegend, USA (Supplementary Data 1).

### Extraction and assay kits

Total RNA was extracted using a FavorPrepTM Blood/Cultured Cell Total RNA Mini Kit (Favorgen Biotech, China). Genomic DNA was prepared by using a FavorPrep Tissue Genomic DNA Extraction Mini Kit (Favorgen Biotech, China). For Western blotting, signals were visualized by using a SuperSignal West Femto Maximum Sensitivity Substrate Kit (Thermo, USA). Lymphocyte isolation was performed with a Ficoll density gradient centrifugation kit (GE Healthcare, USA). Cell-free DNA was isolated and quantified by using a Dynabeads® SILANE Viral NA Kit (Thermo, USA) and Quant-iT™ PicoGreen ® dsDNA Reagent and Kits (Thermo, USA).

Reagents and services: LipofectamineTM 3000 (Thermo, USA); TREX1 siRNA (Santa Cruz, USA); cGAS siRNA (Santa Cruz, USA); siRNA control plasmid (Sigma, USA); *Mycobacterium tuberculosis* (*M. tuberculosis*; Difco, USA); mineral oil (Sigma, USA); methotrexate (MTX) (LC labs, USA); Cre adeno-associated virus construction (Ubigene, China); TREX1 adeno-associated virus construction (Ubigene, China); dimethyl sulfoxide (DMSO; ACROS, USA); DAPI (Invitrogen, USA); RIPA buffer (10×, Cell Signaling Technology, USA); EDTA-free protease inhibitors, PhosSTOP inhibitor (Roche, Basel, Switzerland); TRIzol (Invitrogen, USA); FastStart Universal SYBR Green Master Rox (Roche Diagnostics, USA); Maxima™ H Minus cDNA Synthesis Master Mix (Thermo, USA); PVDF membranes (Bio-Rad, USA).

### Clinical samples

Blood samples were collected from patients with RA, patients with osteoarthritis (OA), and healthy volunteed voluntary consent at Hospital, and approval by the research ethics committee (KY 2021010) in the Affiliated Hospital of Southwest Medical University. All of the epidemiological investigations and classification of the volunteerers upon the provision of written informs were carried out according to the American College of Rheumatology criteria. The clinically relevant information and data are provided in Table 1 and Fig. 1G.

**Table 1:** RA, OA, and healthy volunteers’ baseline datasheet

	RA (*N* = 39)	OA (*N* = 25)	Healthy (*N* = 25)
Age (year(mean (range)))	53 (30~73)	57 (30~72)	45 (24~74)
Sex (N(F/M))	24/15	19/6	20/5
Disease duration (year(mean(range))	5 (1~20)	8 (1~23)	NA
CRP (mg/L(mean(range)))	20.2 (1.4~93)	14.5 (3.13~39.5)	NA
ESR (mm/h(mean(range)))	49.5 (8~120)	22.3 (8~47)	NA
**RF (**>20 IU/mL)	31/38	NA	NA

*CRP* C-reactive protein, *ESR* erythrocyte sedimentation rate, *RF* rheumatoid factors, *NA* not assessed.

### DNA fragment preparation and DNA damage model

RA-FLSs were cultured in 6 cm dishes to a confluence of 60–80%. The cells were then exposed to UV light for 5, 10, and 15 min. The medium was removed and washed twice with PBS for the following experiment. RA-FLSs were harvested for DNA extraction using a DNA extraction kit (Favorgen). DNA fragmentation was performed by ultrasonication for 30 min to shear the DNA into fragments of 500 bp as determined by DNA gel electrophoresis. RA-FLSs cultured in 6-well plates were transfected with 5 μg of DNA fragments for 24–72 h in the absence or presence of Lipofectamine 3000. Furthermore, another group of RA-FLSs was also transfected with different concentrations of DNA fragments (1, 5, and 10 μg) with Lipofectamine 3000 for 24 h. All the medium was removed, and the cells were washed once with PBS before the next step of analysis.

### siRNA transfection

The selected RA-FLSs were transfected using a Lipofectamine 3000 kit (Thermo, USA) according to the manufacturer’s protocol. Briefly, after the cells were cultured in a plate overnight to a confluence of 60–80%, the medium was aspirated and washed once with PBS. The TREX1 or cGAS siRNA plasmid for knockdown of TREX1 or cGAS was mixed with 250 μl of Lipofectamine 3000 and incubated for 15 min. Then, this mixture was mixed with Opti-MEM in a total volume of 1 ml and added to a 6-well plate for incubation for 12 h. After incubation for 4 h, another 1 ml of medium was added to the wells and incubated for 24 h. After 48 h, the cells were transfected with DNA fragments for 24, 48 and 72 h. Real-time PCR was used to verify the mRNA expression of TREX1, cGAS, and inflammatory cytokines.

### Gene expression analysis by real-time PCR

Total RNA from blood and cultured cells was isolated using the FavorPrepTM Blood/Cultured Cell Total RNA Mini Kit (Favorgen Biotech) according to the manufacturer’s protocol. Total RNA from frozen animal synovium and tissues was extracted with TRIzol (Invitrogen, USA). Approximately 20–40 mg of frozen tissue was mixed with 1 ml of TRIzol reagent and homogenized by at least 3 cycles in a homogenizer. Then, 100 μl of chloroform was added and shaken vigorously for 5 min, and the mixture was centrifuged at 4 °C and 12,000 × *g* for 15 min. The supernatant from the phenol/chloroform extraction step was collected (approximately 400–500 µl) and precipitated with isopropanol (1:1.2 supernatant:isopropanol) for 30 min on ice; this mixture was then centrifuged at 12,000 × *g* for 15 min. The precipitate was washed with 70–80% cold ethanol. The white pellet was completely dissolved in 50 µl of RNase-free water. A UV spectrophotometer (NanoDrop Technologies, USA) was used to measure the quality and concentration of RNA.

For real-time PCR analysis, 1 μg of RNA was reverse transcribed with Maxima™ H Minus cDNA Synthesis Master Mix (Thermo, USA). The mixture was incubated at 25 °C for 10 min and at 50 °C for 15 min, and the reaction was then terminated by heating at 85 °C for 5 min. The PCR mixture (total 20 µl) comprised 0.5 µl of template DNA, 0.5 µl each of the forward and reverse primers, 0.5 µl of template, 10 µl of SYBR Master Mix (Roche Diagnostics, USA), and 8.5 µl of ddH2O to bring the total volume up to 20 µl. Quantification of gene expression was performed with a ViiA 7 Real-Time PCR System (Applied Biosystems). All mRNA expression data were normalized to the reference gene β-actin using the ΔΔCT method for relative quantification (Supplementary Data 1).

### Western blotting

RA-FLSs incubated with DNA fragments in the absence or presence of Lipofectamine 3000 were added to RIPA lysis buffer for protein lysate preparation. Proteins in the centrifuged supernatant were then separated by 10% SDS–PAGE. The proteins on the PAGE were transferred to a PVDF membrane, which was probed first with antibodies against TREX1 (1:1000), cGAS (1:1000), E2F1 (1:1000) and β-actin (1:1000) and then with HRP-conjugated goat anti-rabbit IgG as the secondary antibody (1:5000). The immunoreactive bands were then visualized by ECL with a FluorChem R system (ProteinSimple, America). Quantitative analysis of Western blot signals was performed using ImageJ software.

### SD rats used for establishment of the experimental arthritis model (AIA) and treatment

Wild-type male Sprague–Dawley (SD) rats weighing 110–120 g were purchased from Beijing Vital River Laboratory Animal Technology (stock# 101). Conditional knockout (Cko) TREX1^+/-^ SD rats were generated using CRISPR/Cas9 by Cyagen (GuangZhou, China). LoxP-E2-loxP-mediated knockout of TREX1 in SD rats was accomplished by CRISPR/Cas-mediated genome editing from Cyagen. The rat TREX1 gene (GenBank accession number: NM_001024989.1, Gene ID: 100049583) is located on chromosome 8. Two exons have been identified, with the ATG start codon and TAA stop codon in exon 2. Exon 2 was thus selected as the region for conditional knockout. Deletion of exon 2 was expected to result in loss of function of the rat Trex1 gene by inducing a frameshift of downstream exons. The loxP sequence was inserted into the donor vector upstream and downstream of exon 2 by homology-directed repair. gRNA targeting vectors were constructed and confirmed by sequencing, and a donor vector with flanking of homologous arms was also constructed. Cas9 mRNA and gRNA generated by in vitro transcription were coinjected with the donor vector into fertilized eggs to obtain KI rats. The pups were genotyped by PCR, and sequence analysis was then performed.

Rats were bred and maintained at the State Key Laboratory of Quality Research in Chinese Medicines of Macau University of Science and Technology. The genotypes of TREX1 transgenic rats were identified by PCR of tail genomic DNA using two pairs of primers. The animal study was conducted according to the guidelines of the Institutional Animal Care and Use Committee of Macau University of Science and Technology and approved by the Ethics Committee of Macau University of Science and Technology (protocol code MUSTARE – 003 – 2020) on 23 Feb 2020. The experimental/control animals were co-housed and bred separately, and all rats were bred, housed and used under specific pathogen-free conditions in the animal facility of Macau University of Science and Technology. We generally use two methods for animal euthanasia, carbon dioxide (CO_2_) euthanasia (above ten days of age) or injectable agents (barbiturates).

### Model 1 (Inflammatory potency of DNA fragment injection in the AIA model)

SD rats were divided into six groups (*n* = 6~8 rats/group) as follows: (1) healthy control (*n* = 7), (2) AIA model (*n* = 8), (3) AIA + DNA (200 μg; *n* = 7), (4) AIA + DNA (50 μg; *n* = 7), (5) healthy control + DNA (200 μg; *n* = 7), and (6) healthy control + DNA (50 μg; *n* = 7). Adjuvant arthritis was produced by intradermal administration of CFA H37Ra to rats. The adjuvant containing the dried, heat-killed strain of mycobacterium tuberculosis (Difco, USA, 10218823) was dissolved in mineral oil (Sigma, USA, 8042-47-5) with concentration 2.5 mg/ml. After slow grinding in the same direction, the adjuvant was then injected through the tail root of the rat. After that, DNA fragments were injected into the knee joints of rats, and 0.1 ml of the above mixture was then injected subcutaneously at the base of the tail on Day 0. Arthritis scores and hind paw volumes were evaluated and recorded in every 3 days until Day 30.

### Model 2 (monitoring proinflammatory cytokine levels in SD rats injected with DNA fragments)

SD rats were injected with 100 μg of DNA fragments via the tail vein. Blood samples were collected at 0 min, 30 min, 2 h, 4 h, 24 h, 48 h, and 72 h for proinflammatory cytokine detection using RT–PCR.

### Model 3 (Pharmacokinetic study of serum cfDNA after the tail vein injection of DNA fragments in SD rats)

SD rats were divided into 3 groups (*n* = 6) and tail vein injected with 50, 100 and 200 μg of DNA fragments, followed by blood sampling through the orbit at the following 5 time points: 0, 4, 24, 48 and 72 h. Serum cfDNA concentrations were measured using the Quant-iT™ PicoGreen ® dsDNA Reagent. By comparing the concentration of cfDNA in serum of RA patients with that of SD rats, the optimal tail vein DNA fragmentation injection concentration and time points in SD rats were selected.

### Model 4 (monitoring proinflammatory cytokine levels in AIA rats injected with DNA fragments after the first appearance of hind paw swelling)

SD rats were divided into 4 groups (*n* = 6 rats/group) as follows: (1) healthy control, (2) AIA model, (3) AIA + DNA (100 μg), and (4) AIA + DNA (50 μg). Mineral oil (Sigma) containing 2.5 mg/ml *M. tuberculosis* (Difco, USA) was ground completely until the mixture turned white, indicating that it was ready for AIA model injection. The SD rats were then injected with complete adjuvant for arthritis induction (on Day 0). In addition, the AIA rats were tail-vein injected with 50 or 100 μg of DNA fragments (sonicated DNA from rat-derived muscle tissue) on Day 0 and continuing in every 2 days until the day (Day *) when hind paw swelling first became visible. Blood samples were then collected on day (*) for proinflammatory cytokine detection using RT–PCR.

### Model 5 (evaluating the anti-inflammatory effect of TREX1 overexpression in AIA rats)

SD rats were divided into 6 groups (*n* = 8 rats/group) as follows: (1) healthy control, (2) AIA (adeno-associated virus, AAV-EGFP), (3) AIA + methotrexate (MTX; 7.6 mg/kg/week), (4) AIA + AAV-TREX1 (tail-vein injection with 1 × 10^11^ PFU), (5) AIA + AAV-TREX1 (tail-vein injection with 1 × 10^9^ PFU), (6) AIA + AAV-TREX1 (joint injection with 2.5 × 10^10^ PFU) and (7) AIA + AAV-TREX1 (joint injection with 2.5 × 10^8^ PFU). Tail-vein injection of AAV-TREX1 (1 × 10^11^ PFU) and AAV-TREX1 (1 × 10^9^ PFU) was conducted 10 days before AIA induction, and joint injection of AAV-TREX1 (2.5 × 10^10^ PFU) and AAV-TREX1 (2.5 × 10^8^ PFU) was performed 10 days before AIA induction. The SD rats that received AAV injection were subsequently injected subcutaneously with 0.1 ml of the mixture at the base of the tail on Day 0. Arthritis scores and hind paw volumes were evaluated and recorded in every 3 days until Day 30.

### Joint swelling measurement and clinical scoring

Joint swelling and clinical scores were evaluated daily from the first injection (Day 0 for rats) until the animals were euthanized. The investigator was blinded to the group allocation when measuring joint swelling. In the AIA model, joint swelling in the rats was evaluated by measuring the volume of the hind paws with a plethysmometer (Ugo Basile, Italy; or Kent Scientific Corp, Connecticut, USA) and calculating the average volume every 3 days. Animals that died during the experimental period were excluded from the analysis. Clinical scores for each hind paw and fore paw of the rats were determined following the standard evaluation process for clinical scoring: the severity of arthritis can be objectively inspected in four paws and is scored for each paw on a scale of 0~4 according to the arthritis index described in Table 2.

**Table 2:** Rat arthritic index

Score	Symptom
0	There was no swelling and erythema evidence on the joint, including the small joints of the front foot and phalangeal joint, large joints including wrist and ankle.
1	There was mild swelling and erythema on ankle.
2	Mild swelling and erythema extended to small joint.
3	There was serious swelling and erythema on large joint.
4	Serious swelling and erythema encompassing on small and large joint.

### Micro-CT analysis

At the end of the treatment period, the rats were humanly euthanized, and the left hind paw of each rat was amputated, fixed with 4% PFA, and then scanned using an in vivo Micro-CT scanner (SkyScan 1176, Bruker, Belgium). The following scanning parameters were used to obtain high-quality images of the joints of the rats: 35 µm resolution, 85 kV tube voltage, 385 µA tube current, 65 ms exposure time, rotation step over 360° 0.7, and a 1 mm Al filter. The images were reconstructed using NRecon software (Bruker Micro CT, Belgium). The Micro-CT score was obtained from five disease-related indices for Micro-CT analysis of the calcaneus: bone mineral density, bone volume fraction, cortical mineral density, trabecular number, and total porosity.

### Cell-free DNA (cfDNA) extraction and measurement

For extraction of cfDNA in the plasma of rats, peripheral blood samples of rats were collected on Day 30 in EDTA-containing tubes. Then, the blood samples were centrifuged at 400 × *g* for 10 min at 4 °C, and the plasma fraction was recentrifuged at 12,000 × *g* for 10 min at 4 °C to remove cell debris and was then stored at −80 °C for analysis. cfDNA was extracted from 100 μL of plasma using a Dynabeads® SILANE Viral NA Kit. The concentration of cfDNA was determined with Quant-iT™ PicoGreen ® dsDNA Reagent and Kits.

### Haematoxylin and eosin (H&E) staining and immunohistochemistry (IHC) of rat tissue samples

Experimental AIA rats were euthanized on Day 30, and the synovium and organs, including the brain, heart, kidneys, liver, spleen, thymus, and joint tissues, were collected for weighing and H&E staining. Synovial samples were immediately frozen in liquid nitrogen and stored at −80 °C. Organs and joint tissues were fixed with 4% paraformaldehyde for 24 h and were then dehydrated and embedded in paraffin blocks at 60 °C. Six-micrometre-thick sections were dehydrated, deparaffinized, and rehydrated according to standard protocols. Synovial, joint, and organ sections were incubated with the anti-TREX1 primary antibody (1:200, Novus Biologicals, USA) at 4 °C overnight. After washing, the slides were incubated with streptavidin-conjugated horseradish peroxidase (BioSite Histo Plus (HRP) polymer anti-rabbit kit; Nordic BioSite, Sweden) for 1 h. They were then mounted with FluorSave™ Reagent (Millipore, USA). Images were acquired by light microscopy (Leica DM2500, Germany). Joint and organ sections from AIA rats were subjected to H&E staining. Images were acquired by light microscopy (Leica DM2500, Germany).

### Genotyping of TREX1-deficient rats

Genomic DNA was isolated from rat tails. Genotyping of TREX1 was performed by polymerase chain reaction (PCR). PCR amplification was performed using primers. Primer sequences: F-AGAGACTCACGGGCTTGTTTGAATC, R- GAGAGTAAGGCAGGCCAGTGGC. Each 25 μL PCR mixture consisted of 4 μL of genomic DNA (~XX μg), 1 μL of each primer (10 μmol/L), 12.5 μL of 2 × Taq PCR Master Mix (constituents: 0.1 U Taq polymerase/μL, 500 μM each dNTP and PCR buffer), and 6.5 μL of ddH2O (DNase/RNase-free). PCR was performed with an initial denaturation step at 95 °C for 5 min, followed by 35 cycles of denaturation at 95 °C for 30 s, annealing at 60 °C for 30 s and elongation at 72 °C for 35 s. The amplified PCR products were subjected to electrophoresis on a 1.0% agarose gel with 0.5 µg/mL ethidium bromide (EB) prior to visualization by ultraviolet light.

### Establishment of the AIA model using Cko TREX1^-/-^ rats

#### Model 1 (Knee joint cavity injection of Cre adenovirus into Cko TREX1^-/-^ rats)

TREX1 conditional knockout (Cko TREX1^-/-^) rats were divided into 6 groups (*n* = 6–7 rats/group) as follows: (1) Cko TREX1^-/-^ (healthy control, *n* = 7), (2) Cko TREX1^-/-^ (AIA, *n* = 6), (3) Cko TREX1^-/-^ (AIA + high concentration (2.5 × 10^10^ PFU) of Cre adenovirus (CreH), *n* = 6), (4) Cko TREX1^-/-^ (AIA + low concentration (2.5 × 10^8^ PFU) of Cre adenovirus (CreL), *n* = 6), (5) Cko TREX1^-/-^ (healthy control + CreH, *n* = 6), and (6) Cko TREX1^-/-^ (healthy control + CreL, *n* = 6). Rats in the three healthy control Cko TREX1^-/-^ groups and three AIA Cko TREX1^-/-^ groups were injected in the knee joints with nucleic acid-free water or DNA fragments (200 μg), respectively, on Day 0. Approximately 10 days before the Cko TREX1^-/-^ rats were injected with complete adjuvant for arthritis induction (on Day 0), they were injected in the knee joints with Cre adenovirus (2.5 × 10^8^ PFU (CreL) or 2.5 × 10^10^ PFU (CreH)) to facilitate knockout of the TREX1 gene.

#### Model 2 (tail-vein injection of Cre adenovirus into Cko TREX1^-/-^ rats)

Cko TREX1^-/-^ rats were divided into 6 groups (*n* = 6~12 rats/group) as follows: (1) Cko TREX1^-/-^ (healthy control, *n* = 7), (2) Cko TREX1^-/-^ (AIA, *n* = 12), (3) Cko TREX1^-/-^ (AIA + CreH, *n* = 7), (4) Cko TREX1^-/-^ (AIA + CreL, *n* = 8), (5) Cko TREX1^-/-^ (healthy control + CreH, *n* = 8), and (6) Cko TREX1^-/-^ (healthy control + CreL, *n* = 6). Rats in the three healthy control Cko TREX1^-/-^ groups and the three AIA Cko TREX1^-/-^ groups were injected via the tail vein with nucleic acid-free water or DNA fragments (100 μg), respectively, on Day 0. Approximately 10 days before the Cko TREX1^-/-^ rats were injected with complete adjuvant for arthritis induction (on Day 0), they were injected via the tail vein with Cre adenovirus (1 × 10^9^ PFU (CreL) or −1 × 10^11^ PFU (CreH)) to facilitate knockout of the TREX1 gene. On Day 30, all rats were euthanized, and blood, organs and joint tissue were collected for biochemical assays.

### Lymphocyte isolation, intracellular cytokine staining, and flow cytometry analysis

Peripheral blood mononuclear cells (PBMCs) were isolated from AIA rats using a standard Ficoll density gradient centrifugation kit (GE Healthcare). T cells were stained as indicated with the following anti-rat CD monoclonal antibodies (mAbs): APC/Cyanine7 CD45, FITC CD3, PerCP/Cyanine5.5 CD4, and APC CD8a. For Foxp3 cell staining, cells were surface stained for CD3, CD4, and CD45; washed, fixed, and permeabilized using a Foxp3 Staining Buffer Set; and then immunostained with PE anti-mouse/rat/human FOXP3 antibodies (all reagents were obtained from BioLegend, Beijing). Cells were acquired using a FACSAria flow cytometer (BD Bioscience) and further analysed using FlowJo v10 software (TreeStar, Inc.). Gating strategies used for flow cytometry was indicated in Supplementary Fig. 12.

### Statistics and reproducibility

For analysis of two groups, Student’s *t* test was used. One-way analysis of variance was used to analyse differences among 3 or more groups. All results are expressed as the means ± SEMs. GraphPad Prism 9.0 software (GraphPad Software, USA) was used for statistical evaluation of the data. *p* < 0.05 was considered significant. No statistical method was used to predetermine sample size; no data were excluded from the analyses; the experiments were randomized; the Investigators were blinded to allocation during experiments and outcome assessment.

### Reporting summary

Further information on research design is available in the Nature Portfolio Reporting Summary linked to this article.

## Supplementary information

Supplementary Information

Description of Additional Supplementary Files

Supplementary Data 1

Reporting Summary

## Data Availability

The data that support the findings of the study are available from the corresponding author upon reasonable request. Source data are provided with this paper.

## Source data

Source Data
