# Supplementary material for: Age-related self-DNA accumulation may accelerate arthritis in rats and in human rheumatoid arthritis
Source: Nat Commun. 2023 Jul 20;14:4394. doi: 10.1038/s41467-023-40113-3 (PMC10359253; doi:10.1038/s41467-023-40113-3)
Supplement: Supplementary file 1 — Supplementary Information [file 41467_2023_40113_MOESM1_ESM.pdf]

Age-related Self-DNA accumulation may accelerate arthritis in rats and in human rheumatoid arthritis

Supporting Information

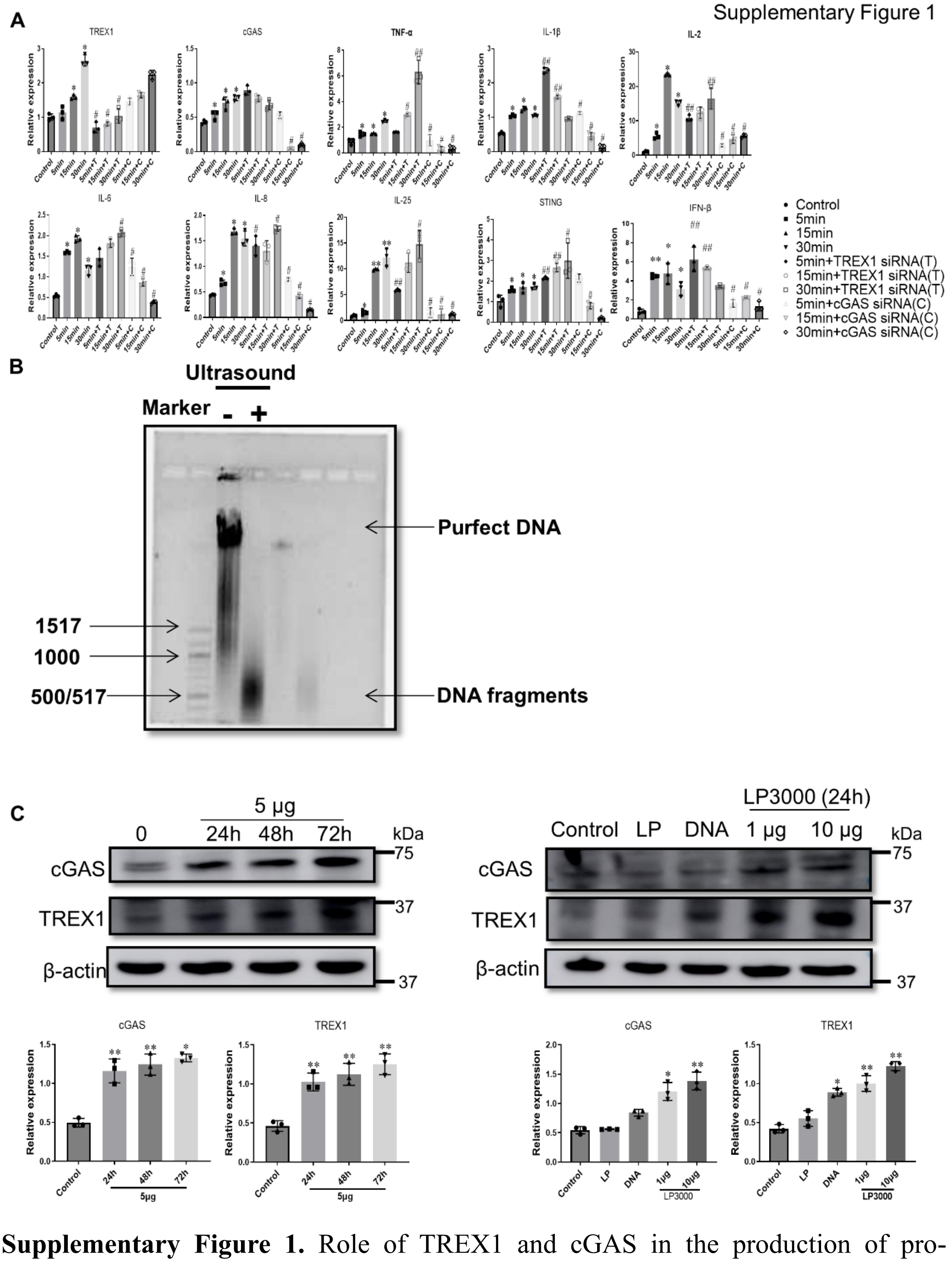

Supplementary Figure 1. Role of TREX1 and cGAS in the production of pro-

inflammatory mediators in RA-FLS exposed with UV light. (**A**) The RA-FLS silenced with siRNA for TREX1 or cGAS were exposed to UV light from 0-30 min, the mRNA of UV-treated cells were then harvested and the gene expression level of pro-inflammatory mediators was detected by RT-PCR. All samples are biologically independent. *P<0.05, **P<0.01 versus control groups, statistical significance was analyzed by one-way ANOVA; for #P<0.05, ##P<0.01 versus UV treatment alone, statistical significance was analyzed by *t*-test. Data are presented as the mean ± s.e.m from three independent experiments. (T, TREX1 siRNA; C, cGAS siRNA). (**B**) Preparation of DNA fragments by ultrasound breaking. RAFLS cells were harvested for DNA extraction using DNA extraction kit (Flavagen). DNA fragmentation was prepared by ultrasound breaking until the size of DNA fragments size reached 500 bp in DNA gel. The samples are biological variability, and data are presented from three independent experiments. (**C**). Time- and dose-dependent protein expression of TREX1 and cGAS in RA-FLSs transfected with DNA fragments. RA-FLSs were either transfected with 5 μg of DNA fragments for 24 to 72 h or transfected with 1 μg or 10 μg of DNA fragments for 24 h. Cell lysates were collected and analysed by Western blotting using antibodies against TREX1 and cGAS (n = 3). The bar charts show the quantitation of target protein expression with respect to actin expression using ImageJ software. All samples are biologically independent, and statistical significance was analyzed by one-way ANOVA, *P < 0.05, **P < 0.01 versus control groups. Data are presented as the mean ± s.e.m from three independent experiments. All samples derive from the same experiment and that gels/blots were processed in parallel.

Supplementary Figure 2

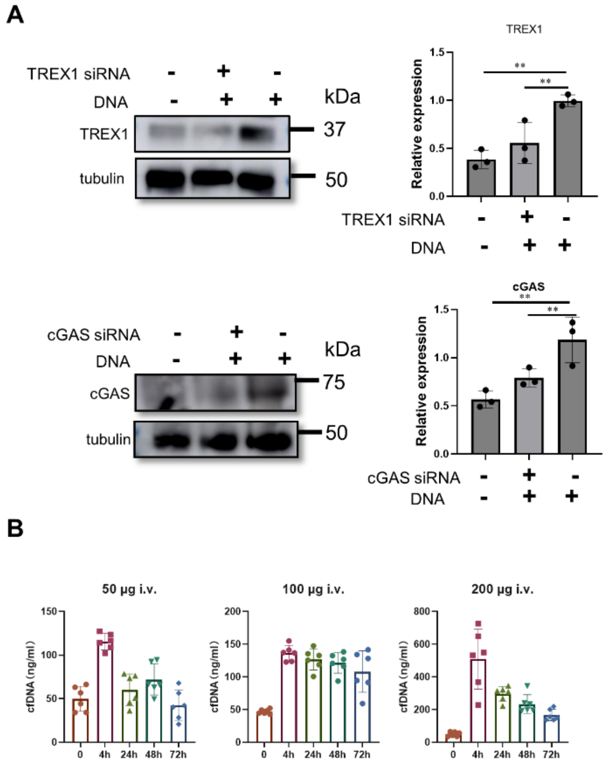

Supplementary Figure 2. (**A**) The protein expression of TREX1 and cGAS after the specific gene knockdown in RAFLS transfected with DNA fragments. RAFLS were transfected with TREX1 siRNA or cGAS siRNA for 24 h prior to the transfection of 5µg DNA fragments. Cell lysates were then collected and analysed by Western blotting using antibodies against TREX1 and cGAS. The quantitation of target proteins was normalized to tubulin. The bar charts show the quantitation of target protein expression with respect to tubulin expression using ImageJ software. All samples are biologically independent, and statistical significance was analyzed by one-way ANOVA, $^{**}P < 0.01$ versus transfection DNA groups. Data are presented as the mean ± s.e.m from three independent experiments. All samples are derived from the same experiment and gels/blots were processed in parallel. (**B**)The pharmacokinetic changes of cfDNA concentrations of blood serum samples in SD rat injected with various concentrations of DNA fragments. SD rats were intravenously injected with 50µg, 100µg, or 200µg of DNA fragments, respectively. Blood serums were collected at 4h, 24h, 48h, and 72h after injection, and the cfDNA concentrations of blood serums were subsequently measured using Quant-iT™ PicoGreen ® dsDNA Reagent. All data are presented as the mean ± s.e.m (n=6).

Supplementary Figure 3

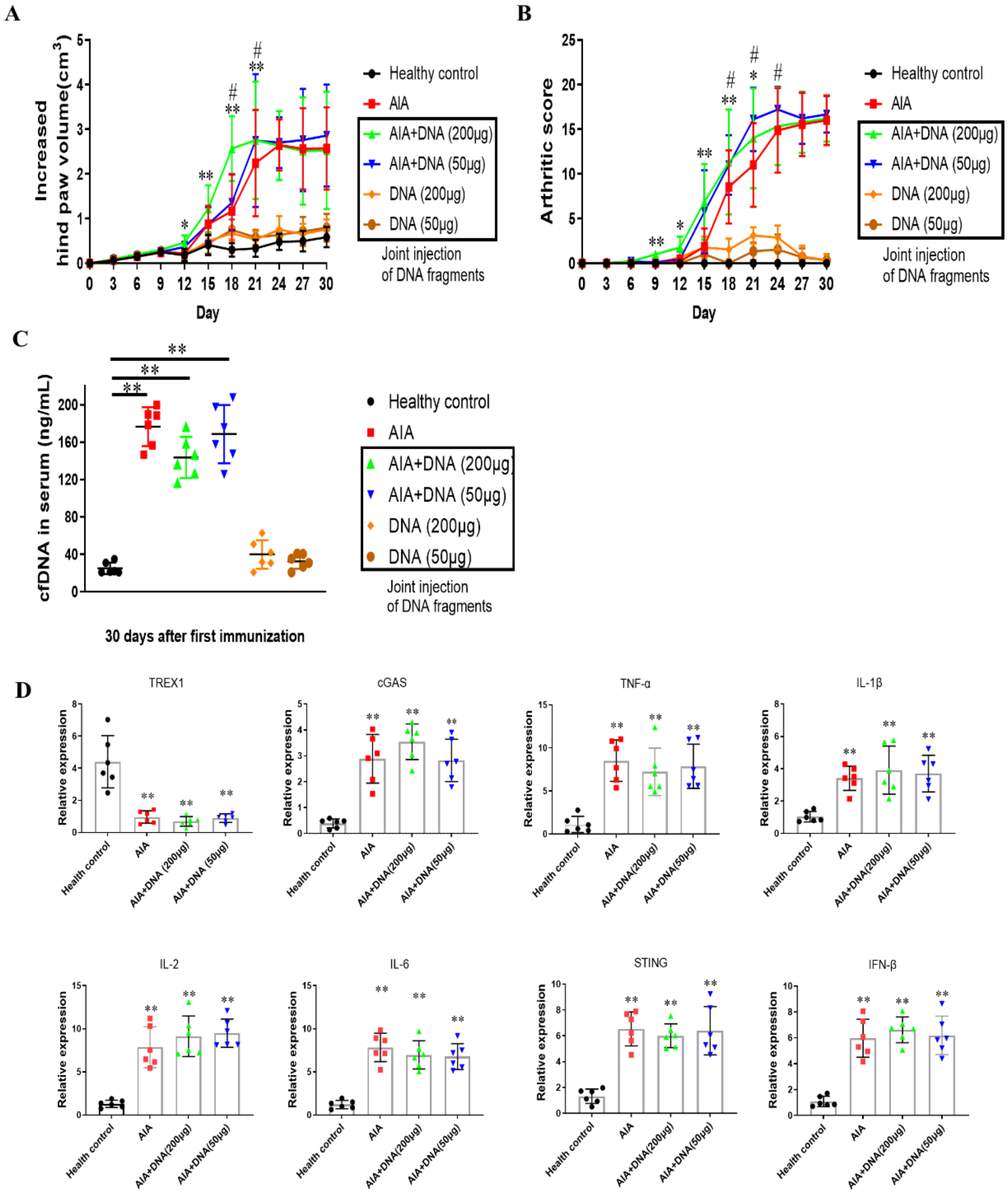

Supplementary Figure 3. The pro-inflammatory effect of joint injected with DNA fragments in AIA rat models. (**A & B**) The hind paw swelling and arthritic scores of DNA fragments-injected AIA rats. Three groups of healthy control rats and three groups of arthritis-induced AIA rats were either injected with nucleic acid free water, DNA fragments (200 μg) or DNA fragments (50 μg) in hint joint region for 30 days, respectively. Hind paw volumes (ml) and arthritic scores were determined in every 3 days. Hind paw swelling was captured at day 30. All samples are biologically independent, and statistical significance was analyzed by t-test, *p<0.05, **p<0.01 for AIA+DNA (200 g) compared with the AIA group, #P<0.05, ##P<0.01 for DNA (200 g) compared with the healthy control group. Data are presented as the mean ± s.e.m (All groups n=9). (**C**) Detection of circulating free DNA (cfDNA) from blood serum of

DNA fragments-injected AIA rats. At day 30, the blood serum was collected for extraction of the cfDNA from all treatment groups using Dynabeads SILANE viral NA. The amount of cfDNA from each treatment group was quantified using Quant-iT^{TM} PicoGreen ® dsDNA reagent and kits. All samples are biologically independent, and statistical significance was analyzed by one-way ANOVA, **$P<0.01$ versus the healthy control group. Data are presented as the mean ± s.e.m. (All groups n=6) (**D**) Gene expression analysis of TREX1 and cGAS signaling pathways in AIA rats injected with or without DNA fragments. PBMCs were harvested from each treatment group for RNA preparation. RT-PCR was adopted to analyze the mRNA expression level of the pro-inflammatory genes. All samples are biologically independent, and statistical significance was analyzed by one-way ANOVA, **$P<0.01$ versus the healthy control group. Data are presented as the mean ± s.e.m (All groups n=6).

Supplementary Figure 4

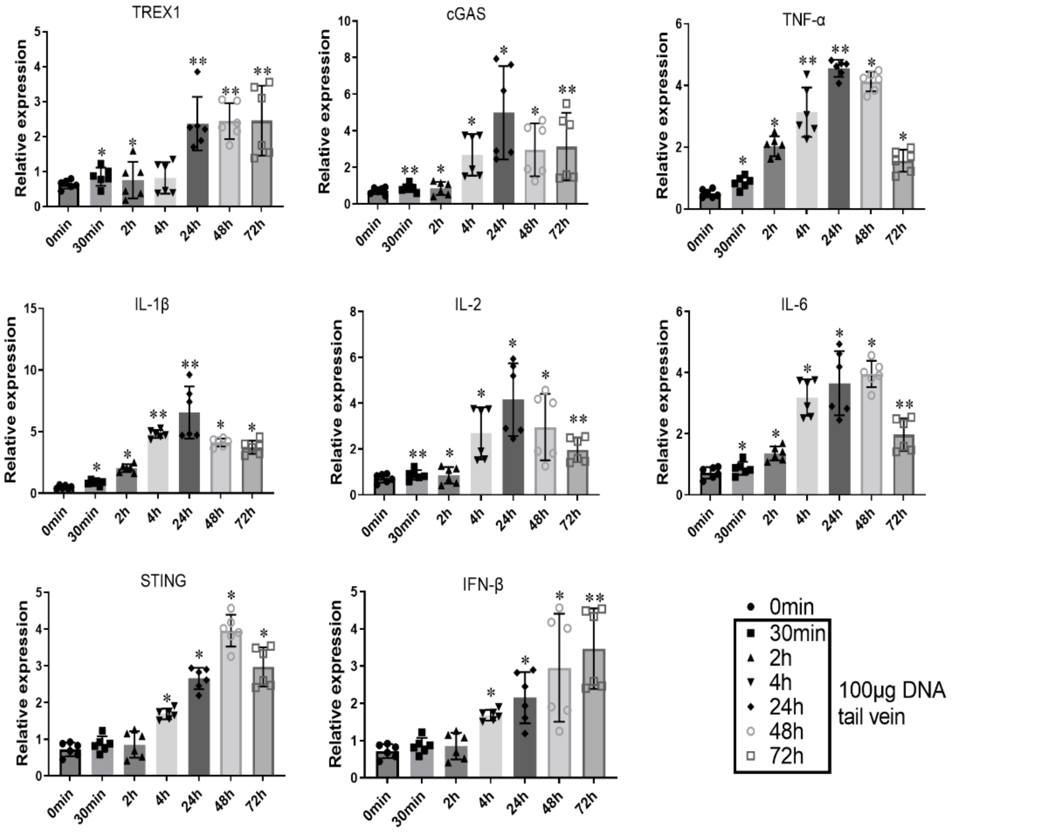

Supplementary Figure 4. The pro-inflammatory effect of DNA fragments on normal SD rats. The healthy SD rats were tail vein injected with 100 μg of DNA fragments (sonicated DNA from dissected rat muscle tissue). At 0 to 72 h after DNA fragments injection, the PBMCs were collected from the DNA fragments-injected SD rats (n = 8) for RNA extraction. Gene expression analysis of TREX1, cGAS, TNF-α, IL-1β, IL-2, IL-6, STING, IFN-β were then validated by RT-PCR. All samples are biologically independent, and statistical significance was analyzed by one-way ANOVA, *P<0.05, **P<0.01 versus the healthy control group. Data are presented as the mean ± s.e.m (All groups n=6).

Supplementary Figure 5-1

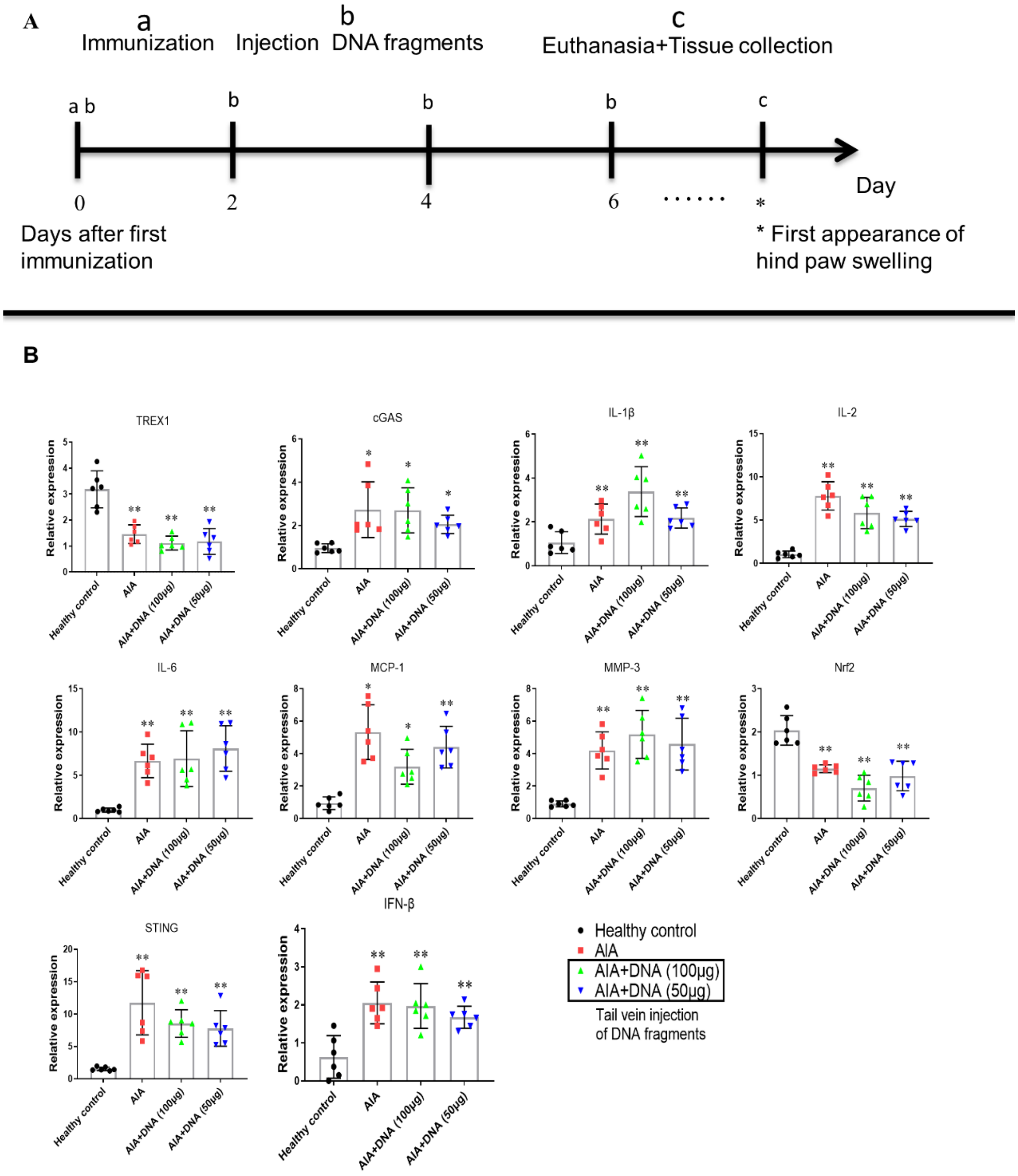

Supplementary Figure 5-2

C

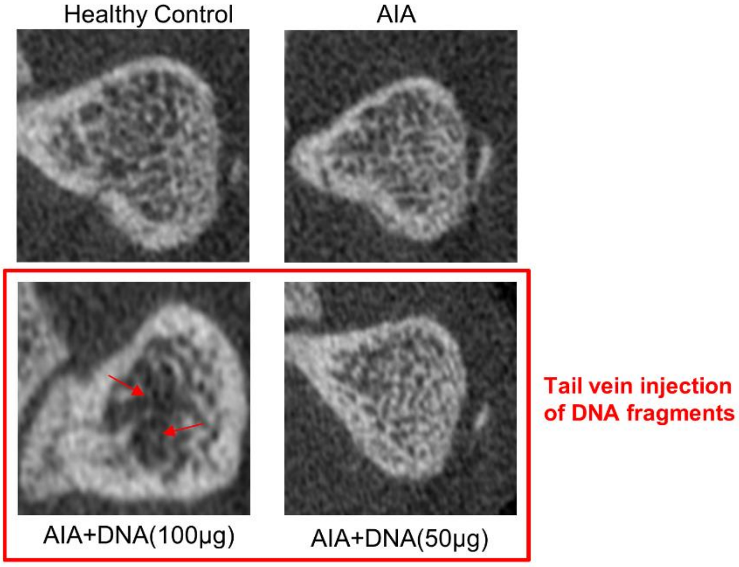

D

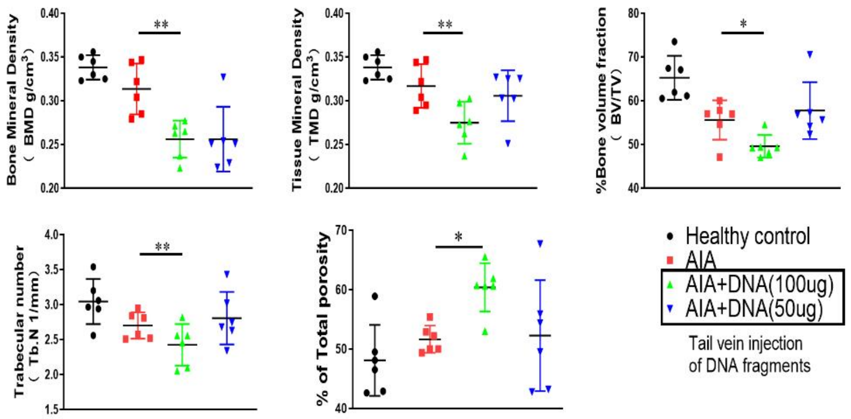

Supplementary Figure 5-1 and 5-2. Correlation of arthritis symptoms and the release of pro-inflammatory cytokines in AIA rat injected with DNA fragments. (**A**) Experimental scheme of the AIA rat models with tail vein DNA injection. After the SD rats were immunized with complete adjuvant for arthritis induction (at day 0), the AIA rats were then tail-vein injected with 50 μg or 100 μg of DNA fragments (sonicated DNA from rat-dissected muscle tissue) from day 0 in every 2 days until the date (*) with first appearance of hind paw swelling. Foot swelling, arthritic score and blood samples were monitored and collected regularly as in the indicated schedule. (a: immunized with complete adjuvant; b: injection DNA fragments; c: euthanasia and tissue collection) (**B**) Gene expression analysis of TREX1-cGAS signaling pathways in AIA rats with tail-vein injection of DNA fragments. At day 12 (*), the AIA rats with hind paw swelling symptoms were sacrificed and PBMCs were collected for RNA extraction and gene expression analysis. The gene expression of cytokines (IL-1β, IL-2, IL-6, MCP-1, MMP-3, Nrf2, STING, IFN-β) were analyzed by RT-PCR. All samples are biologically independent, and statistical significance was analyzed by one-way ANOVA, *P<0.05, **P<0.01 versus the healthy control group. Data are presented as the mean ± s.e.m. (All groups n=6). (**C**) Representative microCT radiographic images

indicating the bone destruction of calcaneus region in the DNA fragments-injected AIA rats with resolution of 19 μm. Red arrows indicate destructive area in hind joint calcaneus region. (**D**) Bone destructive effect in AIA rats with tail-vein injection of DNA fragments. MicroCT analysis of hind joints from DNA fragments-injected AIA rats were conducted to access the bone damaging conditions. MicroCT scores were obtained from five disease-related indexes of bone mineral density (BMD), trabecular number (mm-1) (Tb. N), cortical bone tissue mineral density (g/cm3) (TMD), bone volume fraction (BV/TV), and the percentage of total porosity. All samples are biologically independent, and statistical significance was analyzed by t-tset, *P<0.05, **P<0.01 between the two groups. Data are presented as the mean ± s.e.m. (All groups n=6).

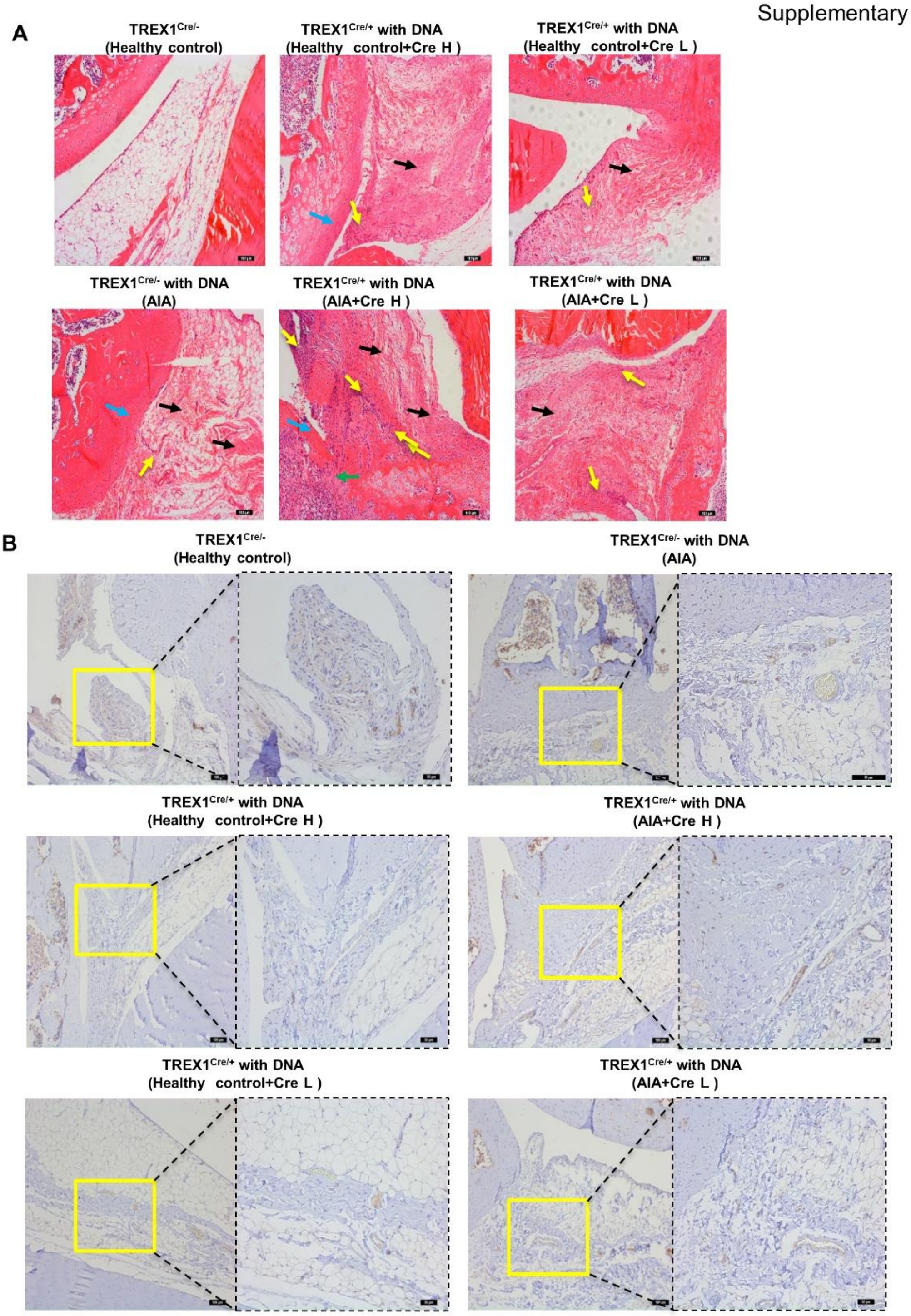

Supplementary Figure 6. Histopathological observations of knee joints in AIA TREX1 conditional knockout (TREX1^{Cre}) rats injected with DNA fragments. (**A**) Representative histological sections of knee joints with H&E staining (×200). Yellow arrows indicate synovial cell proliferation with infiltration of neutrophils, lymphocytes, plasma cells, and other inflammatory cells; black arrows indicate interstitial fibrosis cell proliferation accompanied by neovascularization; blue arrow indicates the thickening of cartilage; green arrows indicate villus-like synovium stretched into the joint cavity, adhered and invaded into the cartilage, and destroyed the articular cartilage

surface. (**B**) Immunohistochemical staining of TREX1 in the knee synovial tissue of AIA TREX1^{Cre} rats (×200). Yellow boxes indicate the keen synovial region. The inset showed the enlarged detail (×400). All the samples are biological variability, and data of A and B are presented from three independent experiments.

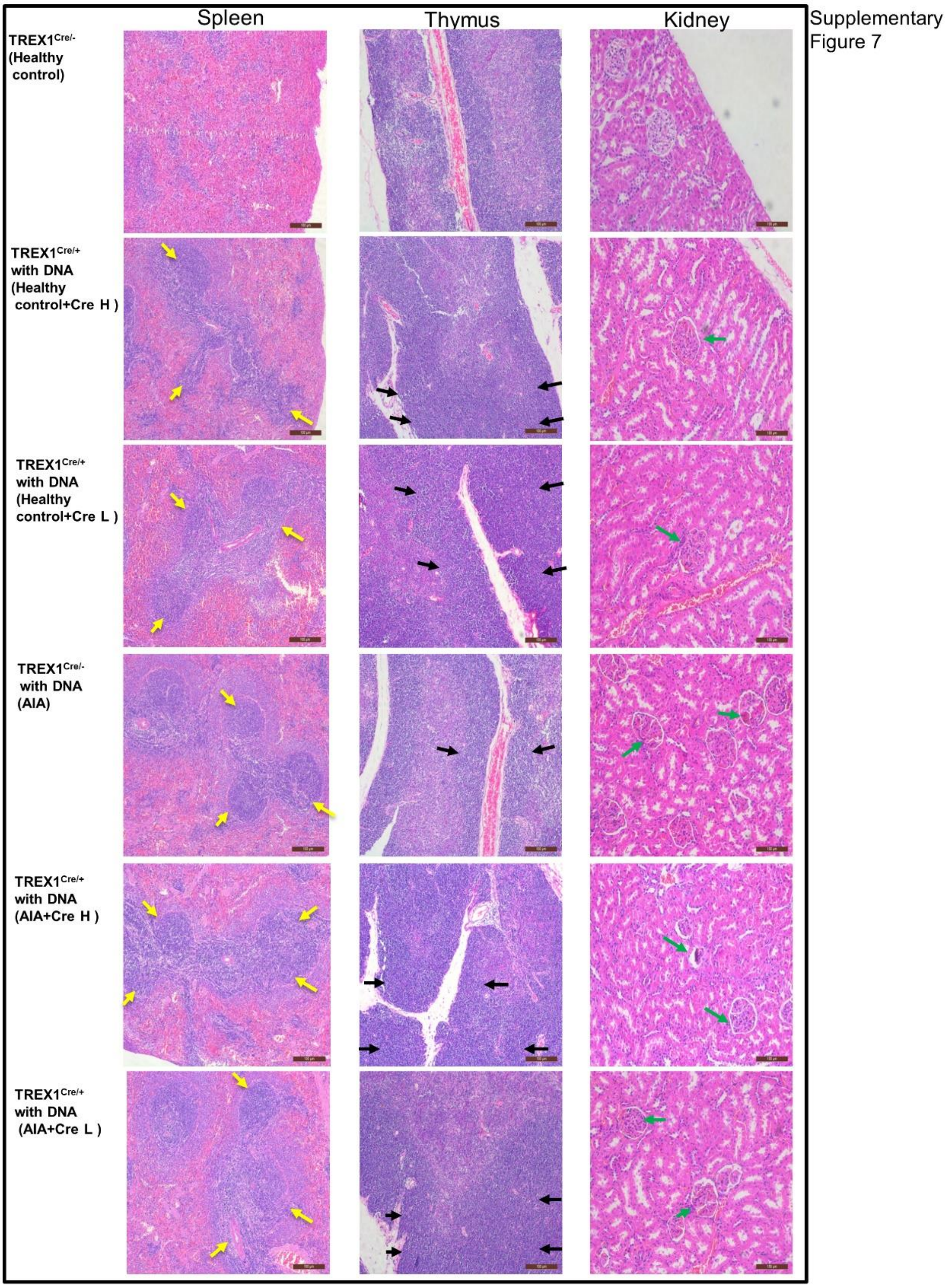

Supplementary Figure 7. Histopathological staining of spleen, thymus and kidney in AIA TREX1^{Cre} rats injected with DNA fragments. Representative histological sections of spleen, thymus and kidney were prepared and stained with H&E (×200). Yellow arrows indicate the white pulps enlargement and fusion, and lymphocyte proliferation; black arrows indicate the clear boundary between thymic cortex and medulla, thickening of cortex and increase of T lymphocytes; green arrows indicate glomerulus edema, unclear structure of glomerulus, and fibrosis. All the samples are biological variability, and in data are presented from three independent experiments.

Supplementary Figure 8

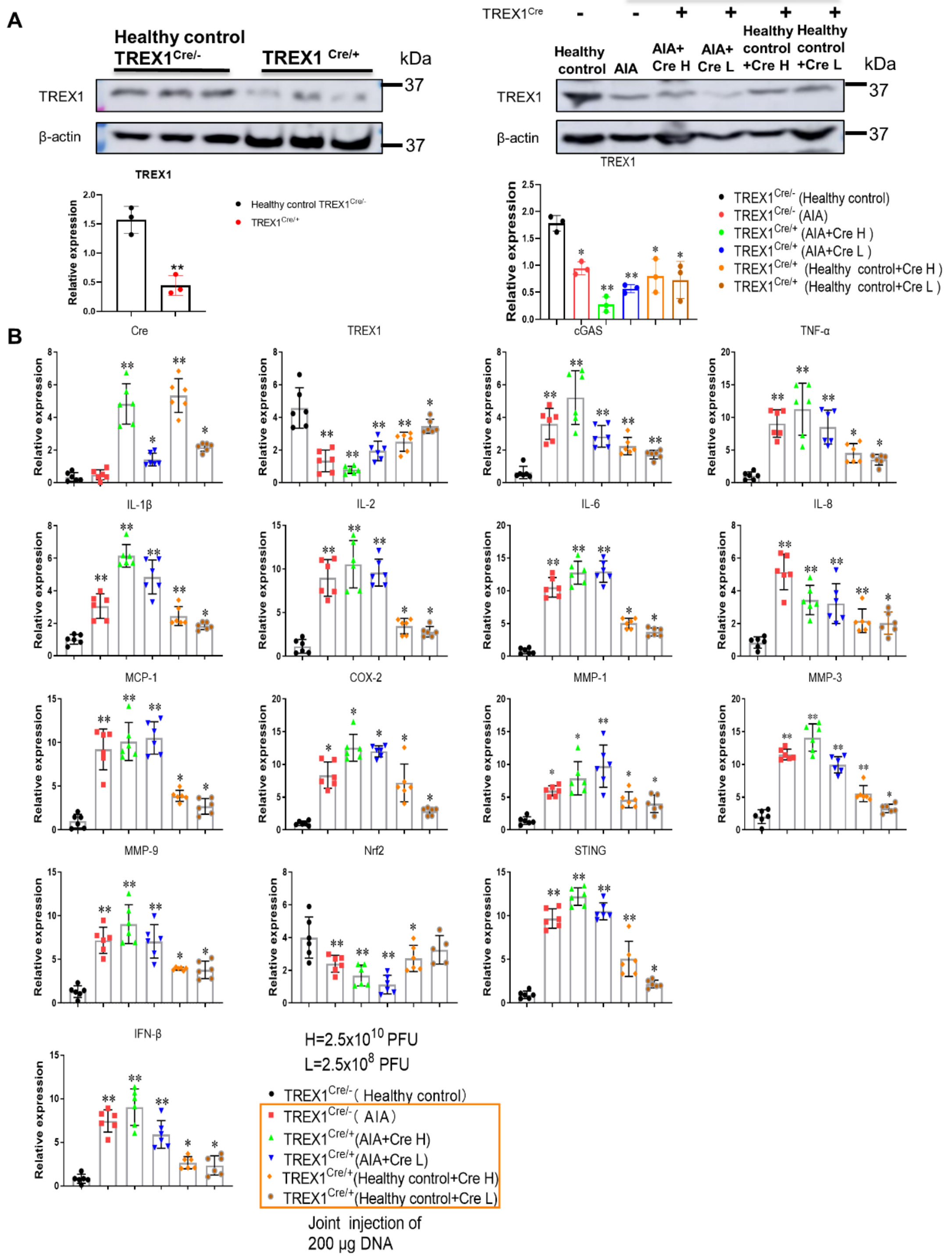

Supplementary Figure 8. Gene expression study of TREX1, cGAS signaling, pro-inflammatory cytokines and metalloproteinase in PBMCs or synovium of DNA fragments-injected AIA TREX1^{Cre} rats. (**A**) Protein expression of TREX1 in AIA TREX1^{Cre} rats. The synovium samples were harvested from all treatment groups for protein extraction and Western blotting using antibody against TREX1 and β-actin (as loading control). Bar charts represent quantitation of TREX1 protein expression. The bar charts show the quantitation of target protein expression with respect to tubulin expression using ImageJ software. All samples are biologically independent, and

statistical significance was analyzed by one-way ANOVA, *P < 0.05, **P < 0.01 versus healthy control. Data are presented as the mean ± s.e.m from three independent experiments. All samples derive from the same experiment and the gels/blots were processed in parallel. (**B**) mRNA expression analysis of TREX1, cGAS signaling, pro-inflammatory cytokines and metalloproteinase. PBMCs samples were collected from all treatment groups for RNA extraction and RT-PCR gene expression analysis using specific primer sets (n = 6-8). All samples are biologically independent, and statistical significance was analyzed by one-way ANOVA, *P<0.05, **P<0.01 versus the healthy control group. Data are presented as the mean ± s.e.m. (All groups n=6).

Supplementary Figure 9

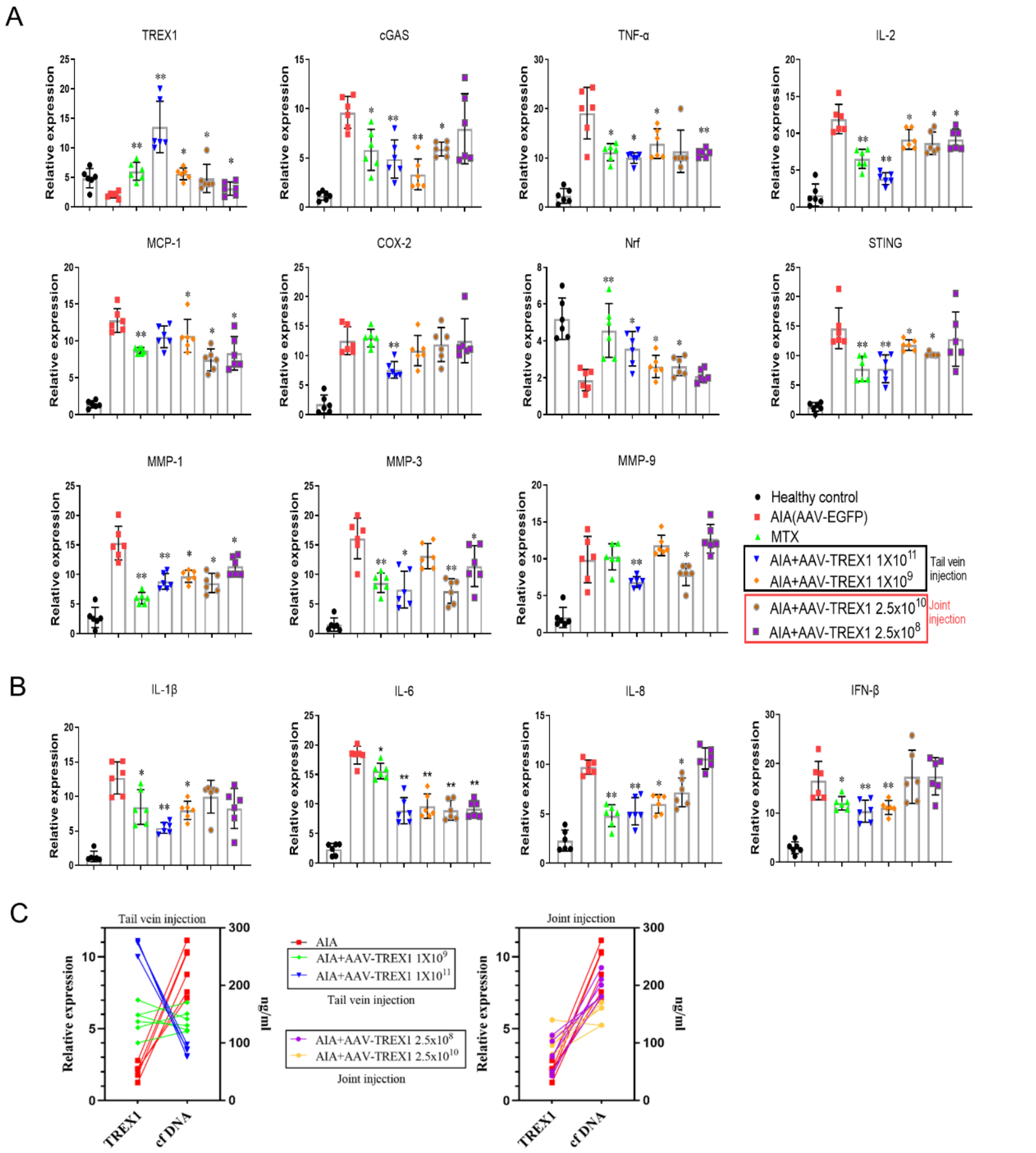

Supplementary Figure 9. Gene expression study of TREX1, cGAS signaling, pro-inflammatory cytokines and metalloproteinase in PBMCs of AAV-TREX1 injected AIA rats. (**A**) mRNA gene expression analysis of TREX1, cGAS signaling, pro-inflammatory cytokines and metalloproteinase. PBMCs samples were collected from all treatment groups for RNA extraction and RT-PCR gene expression analysis using specific primer sets. (**B**) Gene expression analysis of SASP factors in PBMCs of AAV-TREX1 injected AIA rats. In A and B, all samples are biologically independent, and statistical significance was analyzed by one-way ANOVA, $*p<0.05$, $**p<0.01$ versus the AIA group. Data are presented as the mean ± s.e.m. (All groups n=6).(in Figure B ,IL-1β:p=0.0129, p<0.0001, p=0.0016;IL-6:p=0.0080,p<0.0001, p<0.0001, p<0.0001, p<0.0001;IL-8: p<0.0001, p<0.0001, p<0.0001, p= 0.0032;IFN-β: p=0.0208, p=0.0069, p=0.0087) (C) The comparison of TREX1 expression levels with the concentrations of cfDNA in AAV-TREX1 joint- and tail vein-injected AIA rats.

Supplementary Figure 10-1

A

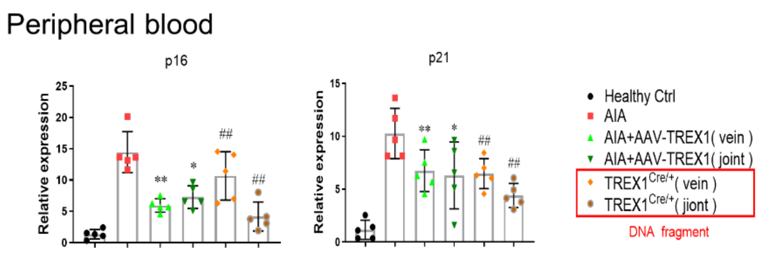

B

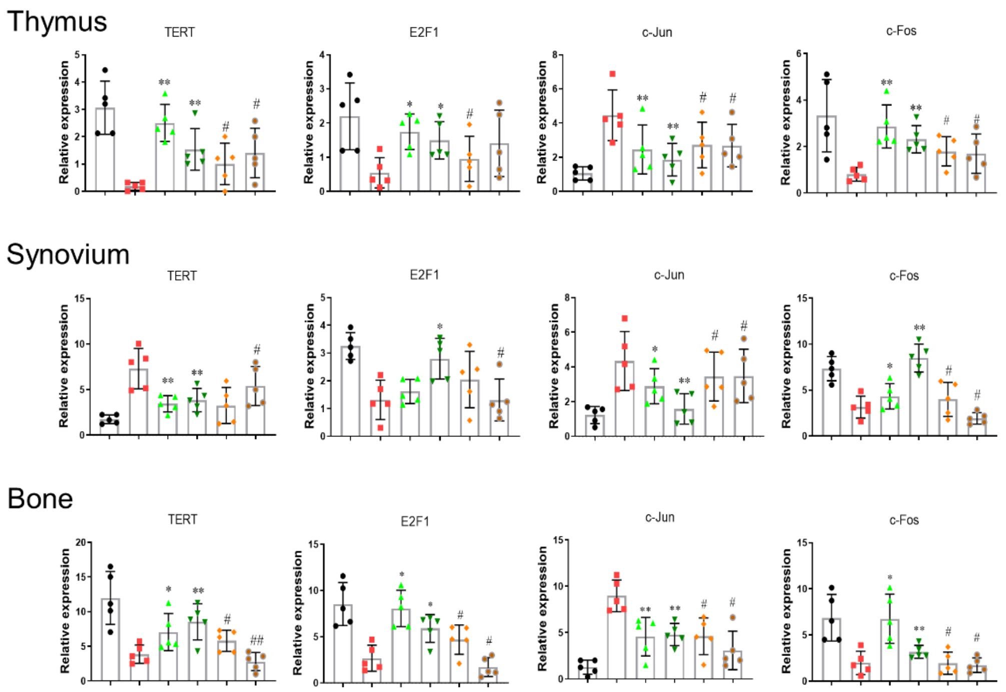

C

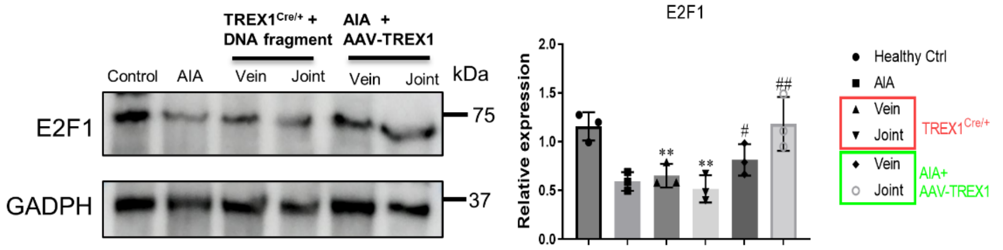

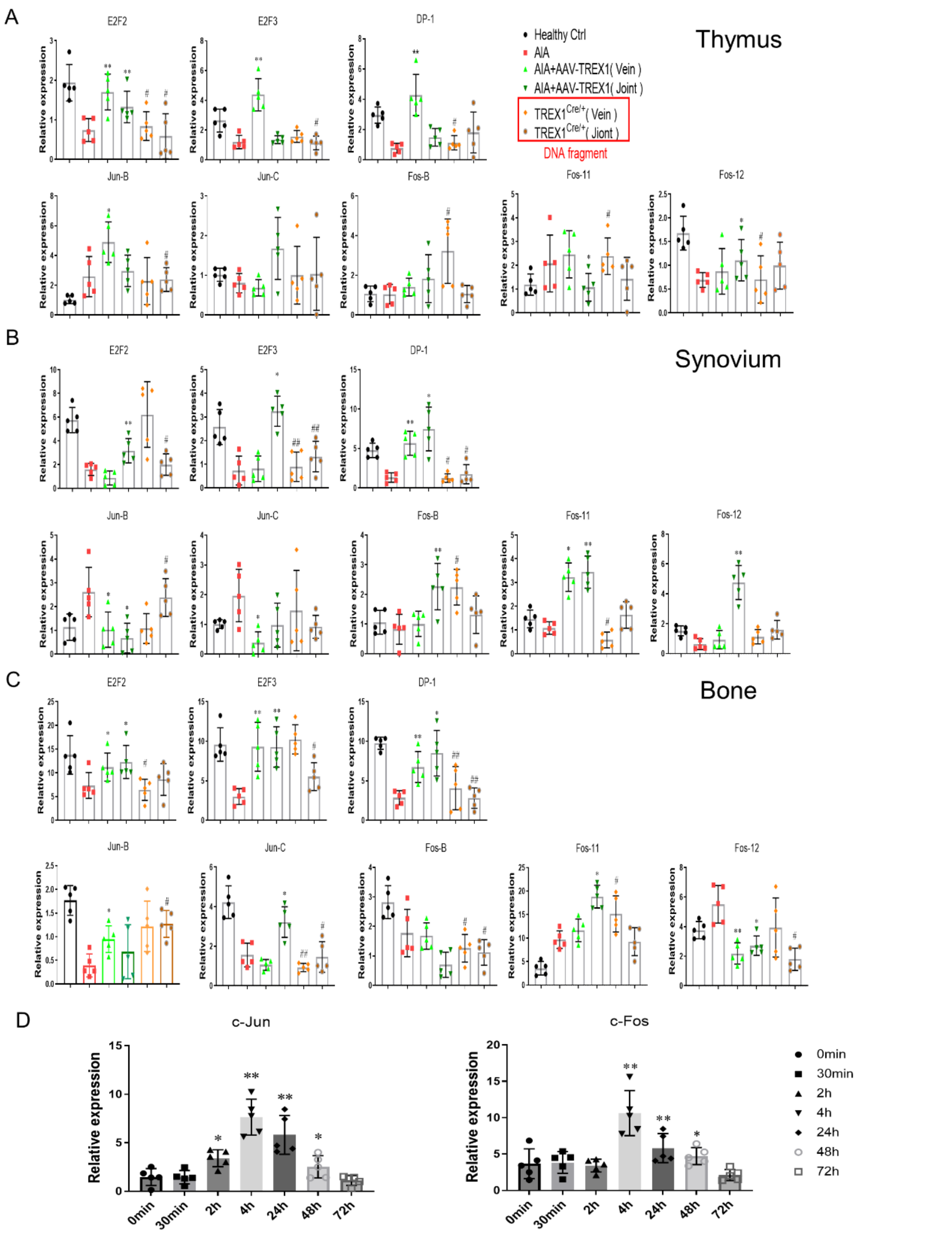

Supplementary Figure 10-1. Study of the transcription factors and tissue specific regulators of TREX1 in AAV-TREX1 injected AIA rats or TREX1^{Cre} rats. (**A, B**) mRNA gene expression analysis of p16, p21, TERT, E2F1, c-Jun and c-Fos. Peripheral blood, thymus, synovium and bone samples were collected from healthy control, AIA model, tail-vein or joint injected AAV-TREX1 AIA rats, and tail-vein or joint injected AAV-Cre TREX1^{Cre} rats for RNA extraction and RT-PCR gene expression analysis using specific primer sets. All samples are biologically independent, and statistical significance was analyzed by one-way ANOVA, *p<0.05, **p<0.01 compared with

AIA model group, #p<0.05, ##p<0.01 compared with healthy control. Data are presented as the mean ± s.e.m. (All groups n=5). (**C**) Expression of E2F1 transcription factor in AAV-Cre injected TREX1^{Cre} rats or AAV-TREX1-injected AIA rats. The synovium tissues were harvested from these treatment groups for protein extraction and Western blotting analysis using antibody against E2F1 and GADPH (as loading control). Bar charts represent the quantitation of E2F1 with reference to actin using Image J software. All samples are biologically independent, and statistical significance was analyzed by one-way ANOVA, **P < 0.01 compared with healthy control groups. #p<0.05, ##p<0.01 compared with compared with AIA groups. Data are presented as the mean ± s.e.m from three independent experiments. All samples were derived from the same experiment and the gels/blots were processed in parallel.

Supplementary Figure 10-2. Study of the transcription factor isoforms of E2F, Jun and Fos families for gene expression regulation of TREX1 in AAV-TREX1-injected AIA rats or TREX1^{Cre} rats. (**A-C**) The mRNA gene expression analysis of E2F2, E2F3, DP-1, Jun-B, Jun-C, Fos-B, Fos-11, Fos-12. Thymus, synovium and bone tissue samples were collected from healthy control, AIA model, tail-vein or joint injected AAV-TREX1 AIA rats, and tail-vein or joint injected AAV-Cre TREX1^{Cre} rats for RNA extraction and RT-PCR gene expression analysis using specific primer sets. All samples are biologically independent, and statistical significance was analyzed by one-way ANOVA, *p<0.05, **p<0.01 compared with 0 min group, #p<0.05 compared with healthy control. Data are presented as the mean ± s.e.m. (All groups n=5). (**D**) The time-dependent expression of c-Jun and c-Fos in DNA fragments-injected normal SD rats. The healthy SD rats were tail-vein injected with 100 µg of DNA fragments (sonicated DNA from rat dissected muscle tissue). The peripheral blood was then collected from the animals (n = 8) at 0 min, 30 min, 2 h, 4 h, 24 h, 48 h and 72 h for RNA extraction. Gene expression analysis of c-Jun and c-Fos were then validated by RT-PCR. All samples are biologically independent, and statistical significance was analyzed by one-way ANOVA, *p<0.05, **p<0.01 compared with 0 min group. Data are presented as the mean ± s.e.m. (All groups n=5)

Supplementary Figure 11

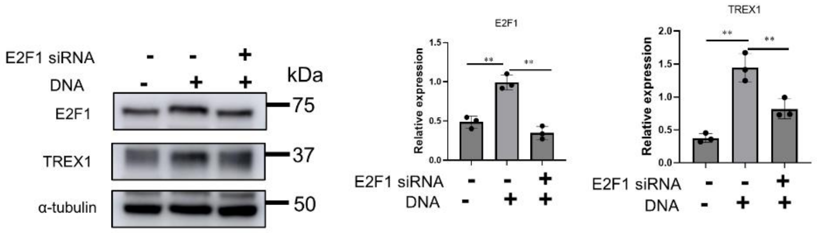

Supplementary Figure 11. The protein expression of E2F1 and TREX1 after the specific gene knockdown in RAFLS transfected with DNA fragments. RAFLS were transfected with E2F1 siRNA for 24 h prior to the transfection of 5μg DNA fragments. Cell lysates were then collected and analyzed by Western blotting using antibodies against E2F1 and TREX1. The quantitation of the target protein was normalized to tubulin. Bar charts represent the quantitation of E2F1 or TREX1 with reference to tubulin using Image J software. All samples are biologically independent, and statistical significance was analyzed by one-way ANOVA, $**p<0.01$ between the two groups. Data are presented as the mean ± s.e.m from three independent experiments. All samples were derived from the same experiment and gels/blots were processed in parallel.

Supplementary Figure 12

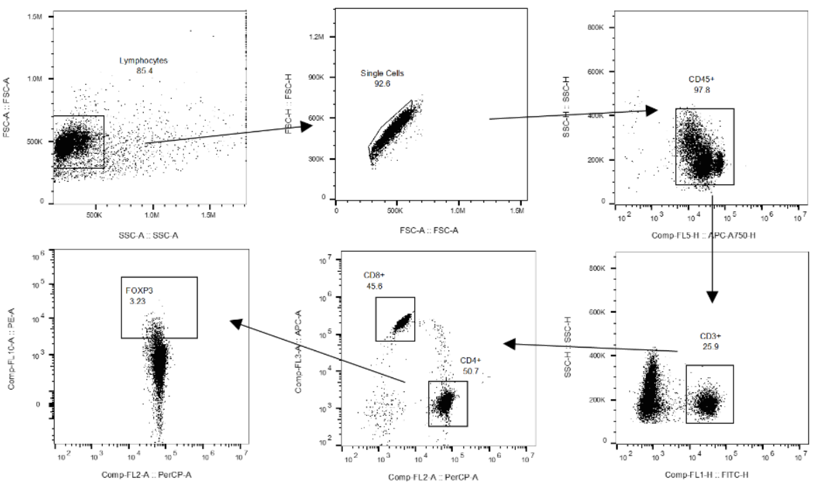

Supplementary Figure 12. Gating strategies used for flow cytometry. Gating strategy to sort CD8（CD45^{+}CD3^{+}CD8^{+}） and Treg（CD45^{+}CD3^{+}CD4^{+}Foxp3^{+}）cells from SD Rat for Immunoscope analysis.

Supplementary Note 1

Figure 1
A: p <0.0001, <0.0001;
B: p <0.0001, <0.0001;
C: p <0.0001, = 0.0002;
D: p <0.0001, <0.0001;
E: p <0.0001, <0.0001;
F: p <0.0001

Figure 2
A:(TREX1:<0.0001, <0.0001, 0.0392; cGAS: <0.0001, 0.0009, 0.0044; TNF-α: <0.0001, 0.0010; IL-1β: <0.0001, 0.0003, 0.0005; IL-2: <0.0001, <0.0001, <0.0001; IL-6: <0.0001, <0.0001, <0.0001; IL-8: <0.0001, <0.0001, <0.0001; IL-25: <0.0001, <0.0001, <0.0001, STING: <0.0001, <0.0001, <0.0001; IFN-β: <0.0001, <0.0001)

B:(TREX1: 0.0026, <0.0001, 0.0392; cGAS: 0.0024, 0.0001, 0.0044; TNF-α: 0.0247, 0.0089, 0.0003, 0.0110, 0.0037, 0.0005; IL-1β: 0.0009, 0.0024, 0.0002, 0.0431; IL-2: <0.0001, <0.0001, <0.0001; IL-6: <0.0001, 0.0003, <0.0001; IL-8: 0.0001, 0.0065, 0.0099; IL-25: 0.0039, 0.0065, STING: 0.0172, <0.0001, <0.0001; IFN-β: <0.0001, <0.0001)

C:(TREX1: 0.0077, <0.0001, 0.0007; cGAS: <0.0001, <0.0001, 0.0003; TNF-α: <0.0001, 0.0010; IL-1β: <0.0001, 0.0003, 0.0005; IL-2: 0.0071, 0.0304, <0.0001, <0.0001, <0.0001, 0.0006; IL-6: 0.0003, 0.0006, <0.0001,<0.0001; IL-8: 0.0210, <0.0001, <0.0001, 0.0146, <0.0001; IL-25: 0.0035, 0.0070, 0.0007, <0.0001, 0.0002 ;STING: <0.0001, 0.0016,0.0004, <0.0001, <0.0001; IFN-β: 0.0483, 0.0006, <0.0001, 0.0003, 0.0003)

Figure 3
A (#:0.0003, 0.0005, 0.0146, 0.0024, 0.0392, 0.0089, 0.0003, 0.0431; *: 0.0005, 0.0146, 0.0039, 0.0065, 0.0009, 0.0044, 0.0003, 0.0005)
B (#:<0.0001, <0.0001, 0.0304, <0.0001, 0.0089, <0.0001, 0.0001, <0.0001;#: <0.0001, 0.0001, <0.0001, <0.0001, 0.0110, 0.0099, <0.0001, <0.0001,)
D (BMD *:0.041, 0.0146, #: <0.0001, 0.0110)
(TMD *:0.0099, #: 0.0431)
(BV/TV*:0.0099, #: 0.0346)
(Tb.n*:0.0024, #: 0.0421)
(Total*:0.0024, #: 0.0361)

Figure 4
A left(*：0.0199，#：<0.0001)
right(*：<0.0001，#：<0.0001)
C left(*:0.0017, <0.0001, <0.0001, <0.0001, 0.0004)

right(*:<0.0001, <0.0001, <0.0001, <0.0001, 0.0001)

Figure 5

A left(#:0.031,*: <0.0001, <0.0001, <0.0001)
right(#:<0.0001,*: 0.00231, 0.00531, <0.0001)

B cfDNA(*:0.0416, <0.0001, 0.0398, 0.3778)

D left (*:<0.0001, <0.0001, 0.0395, 0.0025, 0.0013)
right(*:<0.0001, <0.0001, 0.0267, 0.0190)

Figure 6

A E2F1(*:<0.0001, <0.0001) DP-1(*:<0.0001, <0.0001, <0.0001)
TREX1(*:0.0397, 0.0292, 0.0003)

C E2F1(*:<0.0001, 0.0008) TREX1(*:0.0330, 0.0209)

E E2F1(*:<0.0001) TREX1(*:<0.0001)

F TREX1(*:0.0141, 0.0074) c-Fos(*:0.0140) c-Jun(*:0.0015, 0.0002)

G TREX1(*:0.0087, 0.0020) c-Fos(*:0.0021, 0.0012)
