# Supplementary material for: Age-related self-DNA accumulation may accelerate arthritis in rats and in human rheumatoid arthritis
Source: Nat Commun. 2023 Jul 20;14:4394. doi: 10.1038/s41467-023-40113-3 (PMC10359253; doi:10.1038/s41467-023-40113-3)
Supplement: Supplementary file 2 — Description of Additional Supplementary Files [file 41467_2023_40113_MOESM2_ESM.pdf]

Description of Additional Supplementary Files

File Name: Supplementary Data 1
Description: All the reagents and primers used in this research.
